# Dissociative experiences among Kenyan youth in the Nairobi metropolitan area: Associations with psychosis risk symptoms

**DOI:** 10.1017/gmh.2026.10265

**Published:** 2026-06-25

**Authors:** David Ndetei, Victoria Mutiso, Denis Kioko, Samuel Walusaka, Susan Malinda, Pascalyne Nyamai, Yvonne Kiogora, Kevin Onuonga, Diana Achola, Diana Thakya, Eric Jeremiah, Veronica Onyango, Christine Musyimi, Daniel Mamah

**Affiliations:** 1 Africa Institute of Mental and Brain Health (AFRIMEB), Nairobi, Kenya; 2 Department of Psychiatry, University of Nairobi, Nairobi, Kenya; 3 Washington University School of Medicine, St. Louis, Missouri, USA

**Keywords:** dissociative experiences, WERC-DS, psychosis risk, WERCAP, Maladaptive daydreaming

## Abstract

Dissociative experiences, including perceptual distortions, daydreaming and memory lapses, are clinically relevant and possible correlates and associated features of psychosis risk rather than established prodromal indicators. This study examined dissociative experiences patterns among Kenyan youth and their associations with sociodemographic factors, risk for psychosis and mood disorders. This was a cross-sectional study among youth aged 14–30 years in the Nairobi Metropolitan Area. Participants completed the Washington Early Recognition Center Affectivity and Psychosis Screen (WERCAP) and the Washington Early Recognition Center Dissociation Screen (WERC-DS): Exploratory factor analysis, group comparisons and reliability testing were applied. Dissociative experiences were common among participants, with 25–47% endorsing the highest frequency on individual daydreaming items, and 6.5–39.5% endorsing the highest severity on individual items assessing perceptual distortions. Three factors emerged (Daydream Intensity and Engagement, Perceptual Distortion and Memory Lapses), demonstrating strong internal consistency (*α* = 0.89). Youth at high risk for psychosis reported significantly higher dissociation scores than low-risk peers (*p* < 0.001), specifically perceptual distortions (*p* = 0.001) and immersive daydreaming (*p* < 0.001). Dissociative experiences varied significantly by age and socioeconomic status. Dissociative experiences are prevalent and developmentally patterned among Kenyan youth and are strongly associated with psychosis risk, supporting dissociation as a psychological construct associated with psychosis-risk status in low-resource settings.

## Impact statement

This study provides one of the first large-scale community-based examinations from Africa on the prevalence, structure and clinical relevance of dissociative experiences among youth. The findings contribute to the existing knowledge about the dissociative phenomenon in the non-Western environment and provide strong evidence regarding their correlation with the risk of psychosis. Notably, daydreaming and perceptual distortions were identified as the most prominent and clinically significant dimensions, differentiating high-risk participants and their low-risk counterparts. In contrast, dissociation was not significantly linked to mood disorder risk. These findings have implications for early detection and intervention methods in low-resource African contexts. By defining the presence of dissociative experiences as possible indicators of early detection, the study contributes to the creation of culturally sensitive screening tools and community-based prevention programs. This research provides a groundwork for longitudinal and intervention studies, which can help to screen youth who are at high risk of schizophrenia and enhance mental health outcomes among different African communities.

## Introduction

Dissociative experiences encompass disruptions in consciousness, memory, identity and perception and can range from mild, everyday phenomena to pathological manifestations (Dell, 2009; Lyssenko et al., 2018). Globally, dissociative experiences such as perceptual distortions, intense daydreaming and memory lapses have been closely linked to trauma exposure and disruptions in identity integration (Soffer-Dudek and Somer, 2022). Within this spectrum, maladaptive daydreaming (MD) has emerged as a distinct condition characterized by excessive, immersive fantasy that causes significant distress and functional impairment. A major contribution is the Israeli epidemiological study (Soffer-Dudek and Somer, 2022), which used the Maladaptive Daydreaming Scale-16 (MDS-16) among approximately 1,000 community and student participants. The study reported a self-identified prevalence of 4.2%, particularly among younger adults and students, which decreased to about 2.5% following clinical interviews, highlighting the importance of diagnostic validation. The findings further demonstrated that MD is distinct from normative mind-wandering and attentional difficulties, supporting its classification as a dissociative phenomenon. Together with earlier scale development (Somer et al., 2016) and cross-cultural validation studies (Soffer-Dudek et al., 2021), this evidence supports arguments for the inclusion of MD within dissociative disorders in psychiatric diagnostic frameworks (Soffer-Dudek et al., 2025). In Hungary, a study involving over 700 participants found that individuals reporting MD also exhibited significantly higher dissociative tendencies, particularly in identity confusion and diminished control over mental processes (Sándor et al., 2023). Notably, dissociation mediated the relationship between childhood trauma and MD, suggesting that early adverse experiences may foster both self-detachment and immersive fantasy as coping mechanisms.

Dissociative experiences are not evenly distributed across populations but vary systematically by sociodemographic factors, including gender, age, education and socioeconomic position (Ross et al., 1990; Van IJzendoorn and Schuengel, 1996; Kate et al., 2020; Saini et al., 2024). Low socioeconomic status and poverty-related adversity, often operationalized through wealth indices in low- and middle-income countries, are associated with increased exposure to trauma, chronic stress and reduced access to psychosocial resources, which in turn may elevate vulnerability to dissociative symptoms (Kohl, 2010; Klest, 2012; Boyer et al., 2022).

A systematic review of electrophysiological studies found that individuals experiencing depersonalization and derealization demonstrate altered integration of interceptive and external sensory information, indicating measurable neural correlates of dissociative experiences (Murphy, 2023), thus lending biological support to dissociation-related phenomena, including perceptual distortions and deep absorptive states such as MD.

In Sub-Saharan Africa, dissociative experiences often intersect with culturally embedded explanatory models. In Uganda, a comparative study of individuals identified by traditional healers as “spirit possessed” revealed significantly higher levels of psychoform and somatoform dissociation, alongside greater exposure to potentially traumatic events, compared to “non-possessed” controls (Braitmayer, 2014). Importantly, many individuals interpreted their experiences through cultural and spiritual frameworks rather than trauma-based psychiatric models. Subsequent research indicates that traditional healing practices frequently address relational, ancestral and social disruptions, highlighting culturally grounded pathways for understanding and managing dissociative phenomena (Tobin, 2019).

In Kenya, dissociation has been examined primarily in relation to collective trauma and adolescent development. Studies among survivors of the 1998 U.S. Embassy bombing in Nairobi found dissociative symptoms to be common but largely aligned with culturally acceptable and personality-related responses rather than overt psychopathology (Pfefferbaum et al., 2006). More recent research among Kenyan adolescents demonstrated that dissociation and attachment anxiety mediated the relationship between exposure to multiple potentially traumatic events and somatic symptoms, suggesting that dissociation may function as a psychological coping mechanism in the context of trauma, relational insecurity and identity development (Ferrajao et al., 2024).

Emerging empirical evidence further suggests that dissociative experiences may operate independently of mood pathology (Krause-Utz and Krause-Utz, 2025). Although dissociation often co-occurs with affective disorders in clinical settings, research indicates that dissociative symptoms can constitute a distinct psychological dimension rather than a direct expression of bipolar affective disturbance (Pfefferbaum et al., 2006; Krause-Utz and Krause-Utz, 2025). Recent literature has increasingly examined the relationship between dissociative experiences and psychosis-spectrum phenomena, particularly hallucinations, derealization, altered self-experience and disturbances in reality monitoring (Pienkos et al., 2019; Cernis, 2020). Meta-analytic evidence indicates that dissociative experiences are significantly associated with psychotic-like symptoms across both clinical and non-clinical populations, especially hallucinations and trauma-related psychosis presentations (Cernis, 2020). Trauma-informed models further suggest that dissociation may partly mediate the relationship between childhood adversity and psychotic experiences through disruptions in self-processing, emotional regulation and perceptual integration (Schiavone et al., 2018; Pienkos et al., 2019). However, the precise nature of this relationship remains debated, as dissociation may reflect a co-occurring trauma-related dimension, a phenomenologically overlapping construct, or a vulnerability-associated process rather than a direct predictor of psychotic disorder onset. Importantly, most studies examining dissociation and psychosis have been conducted in Western clinical populations, with limited evidence from African community youth samples.

At the same time, dissociative experiences are classically associated with dissociative disorders, including depersonalization/derealization disorder, dissociative amnesia and dissociative identity disorder. Nevertheless, dissociative phenomena are also frequently observed dimensionally within non-clinical populations and across other psychiatric conditions without necessarily meeting diagnostic thresholds for formal dissociative disorders (Pienkos et al., 2019; Pini et al., 2025). The present study therefore focuses on dimensional dissociative experiences within a community youth population rather than the diagnosis of formal dissociative disorders. This approach was selected because subclinical dissociative experiences, such as immersive daydreaming, perceptual distortions and memory lapses, may still carry developmental and clinical relevance when examined alongside psychosis-risk and mood-related experiences in community settings. Dissociative phenomena such as perceptual alterations, immersive daydreaming and memory lapses tend to co-occur and form a cohesive pattern of experiences, whereas their links to mood symptom severity appear limited or inconsistent across populations. In contrast, some clinical studies report higher dissociation in individuals with mood disorders, particularly when comorbid with trauma exposure, suggesting that early adversity may explain part of the overlap (Şar, 2020; Korkmaz, 2024). This distinction is especially relevant in youth and non-clinical populations, where dissociation may serve as a coping or regulatory mechanism in response to stress, trauma and developmental challenges, rather than an indicator of mood disorder (Lewis-Fernández and Kirmayer, 2019; Pinto, 2022). Collectively, these findings highlight the importance of considering dissociative experiences as an independent construct, particularly in culturally diverse contexts where emotional expression and symptom interpretation may differ from dominant Western psychiatric models.

### Gaps in knowledge

Despite this evidence linking dissociative experiences to trauma, identity disruption and functional impairment, substantial gaps remain in understanding dissociation among youth, particularly in low- and middle-income and non-Western contexts. Most existing research, including studies on MD, focuses on adult, clinical or Western populations, offering limited insight into how dissociative experiences vary across developmental stages and sociocultural settings. Consequently, little is known about demographic differences in dissociation or its potential role as an early marker of vulnerability to severe psychiatric conditions. Moreover, dissociation is often treated as secondary to established diagnoses rather than as an independent construct that may precede disorders such as psychosis and bipolar disorder (Allen et al., 1997). Current measurement approaches further limit understanding by inadequately operationalizing dissociation, particularly immersive daydreaming and phenomenological experiences such as perceptual distortions and memory lapses, contributing to the persistent under-recognition of dissociation as a clinically meaningful construct in youth (Dell and O’Neil, 2010; Somer et al., 2017).

This gap highlights the need for population-based research that systematically examines the patterns, correlates and clinical relevance of dissociative experiences among young people in culturally diverse settings. In response to these gaps, this study aims to examine dissociative experiences as a multidimensional psychological construct among youth aged 14–30 years in Kenya and to examine their relationship with sociodemographic factors and risk for psychosis and mood disorders. In the present study, dissociation is conceptualized primarily as a multidimensional psychological construct that may overlap with, and be associated with, psychosis-risk experiences without implying causal or prodromal status.

### Research questions

1. What types and patterns of dissociative experiences, including perceptual distortions, immersive daydreaming and memory lapses, are observed among Kenyan youth aged 14–30 years in the Nairobi Metropolitan Area?
2. To what extent do dissociative experiences function as a distinct diagnostic entity among Kenyan youth aged 14–30 years in the Nairobi Metropolitan Area?
3. How are dissociative experiences associated with risk for psychosis among Kenyan youth aged 14–30 years in the Nairobi Metropolitan Area?

### Aims

1. To identify and describe the types and patterns of dissociative experiences, including perceptual distortions, immersive daydreaming and memory lapses among youth aged 14–30 years.
2. To examine the association between dissociative experiences and socio-demographic characteristics, including wealth index.
3. To assess the relationship between dissociative experiences and risk for psychiatric disorders, with a particular focus on psychosis and mood disorders, among youth.

## Methods

### Study design and setting

This was a community-based cross-sectional study conducted between 2021 and 2025 in the Nairobi Metropolitan region, Kenya. Data were collected in colleges, universities and surrounding community settings. Nairobi Metropolitan region was purposively selected because it captures substantial social, economic and demographic diversity, spanning urban and peri-urban environments, and is broadly reflective of the Kenyan population. The resulting sample should therefore be interpreted as predominantly student-heavy and urban and peri-urban rather than nationally representative of all Kenyan youth populations. Data collection overlapped with the COVID-19 recovery period and the 2022 Kenyan general election, both of which may have introduced social, political and economic stressors that influenced dissociative experiences, particularly among students and urban youth.

### Participants and sampling

Youth aged 14–30 years were recruited from universities and colleges. Eligibility included youth within the specified age range, able to understand the study and willing to provide informed consent or assent. Participants outside the age range, unable to comprehend the questionnaire due to intoxication or illiteracy, or unwilling to participate were excluded. Signed informed consent was obtained from the parents or guardians of participants under the age of 18. A total of 949 participants completed the assessment. Recruitment reflected a structured convenience sampling approach rather than a probability-based national sampling framework.

### Data collection procedures

Twelve research assistants were recruited and trained to carry out data collection. Community entry was facilitated through county and local administrative structures, with engagement of community leaders using standardized study information scripts. Educational institutions were accessed through their administrations. Participants completed self-administered questionnaires in supervised group sessions of up to 25 individuals. Random allocation to research assistants was achieved using numbered vouchers. Research assistants monitored the sessions to ensure confidentiality, prevent discussion and maintain standardized administration. All questionnaires were completed independently. The screening was done using the Washington Early Recognition Center Affectivity and Psychosis (WERCAP) Screen, a 16-item questionnaire, of which 8 items measure the severity of psychosis risk based on both symptom frequency and effect on functioning. A cut-off score of ≥30 was to be used to preselect potential CHR participants based on a previous validation study in Kenya predicting CHR status.

## Measures

### Sociodemographic characteristics

Participants reported age, gender, education level, religion, employment status and marital status. These variables were used to describe the sample and contextualize the findings.

### Socioeconomic status (wealth index)

Socioeconomic status was measured using a household wealth index based on the Demographic and Health Survey approach. Indicators included household asset ownership, water source, toilet facility, floor material and cooking fuel. Principal component analysis was used to derive a composite index with low and high status.

### The Washington Early Recognition Center Affectivity and Psychosis Screen (WERCAP Screen)

The WERCAP Screen is an 18-item self-report questionnaire developed by Mamah (2011) to assess the severity of mood and psychotic symptoms among adolescents and young adults. The tool is designed to be cross-culturally applicable and evaluates symptoms based on both their frequency of occurrence and their impact on daily functioning. Items 1–8 assess mood lability and affective symptoms (a-WERCAP), while items 9–16 assess psychosis risk (p-WERCAP). Responses are rated using a frequency scale ranging from 0 (no) to 5 (almost always) and, for most items, a functional impact scale ranging from 0 (not at all) to 3 (severely). Total scores are obtained by summing the frequency and functionality ratings, with higher scores indicating greater symptom severity. Among adolescents and young adults, p-WERCAP scores of 30 or higher suggest a high risk for developing a psychotic disorder, while scores between 10 and 29 indicate moderate risk. The WERCAP Screen has demonstrated acceptable psychometric properties and has been validated for use in Kenya, Rwanda and the United States, showing reliability and validity in screening for affectivity and psychosis risk in these populations (Ndetei et al., 2019).

### The Washington Early Recognition Center Dissociation Screen (WERC-DS)

The WERC Dissociation Screen (WERC-DS) is a 20-item self-report screening instrument developed to assess a broad and clinically meaningful range of dissociative experiences in both community and clinical populations. It was created in response to two persistent gaps in existing measures: the limited availability of brief yet comprehensive dissociation screens suitable for cross-cultural use, and the frequent clinical conflation of dissociative symptoms with psychotic phenomena across diverse populations (Van Os et al., 2009; Moskowitz et al., 2011).

The WERC-DS captures multiple core domains of dissociation that are commonly reported in dissociative disorders but are often underassessed by existing tools. These domains include depersonalization and derealization (experiences of detachment from oneself or the surrounding environment), dissociative amnesia and memory lapses, perceptual distortions involving altered sensory experiences without loss of reality testing and absorptive or immersive states. Together, these domains reflect both classical dissociative symptoms and less well-characterized forms of dissociative experience (Spiegel et al., 2011; Soffer-Dudek et al., 2025). The scale also incorporates items related to MD, a pattern of excessive, intense and immersive fantasy activity that can cause distress and functional impairment.

The WERC-DS assesses symptom severity primarily based on the frequency of experiences during the past 3 months. For most items, respondents select one of five frequency-based response options: *no*, *rarely*, *sometimes*, *often* or *almost always.* The sole exception is the first item, which assesses the average number of hours per day spent daydreaming about an imaginary life or event; response options for this item are *0 h*, *<1 h*, *1–3 h*, *4–10 h* and *>10 h.* All items are scored on a 0–4 scale, yielding comparable item-level severity scores across the measure.

MD has increasingly been recognized as overlapping with dissociative absorption and may represent a dissociative subtype or dimension (Somer et al., 2016; Soffer-Dudek and Somer, 2022). Its inclusion allows the WERC-DS to characterize both the prevalence and functional impact of these experiences in community samples. Items are rated on a Likert scale reflecting the frequency or intensity of each experience. Factor-analytic work suggests that responses cluster into meaningful dimensions, such as daydreaming intensity and engagement, perceptual distortion and memory lapses, indicating distinct yet related forms of dissociative phenomena in the general population. These dimensions were intentionally designed to bridge traditional conceptualizations of dissociation with emerging perspectives that extend beyond older instruments (Spiegel et al., 2011).

Compared with widely used measures such as the Dissociative Experiences Scale (DES-II), which assesses absorption, depersonalization/derealization and amnesia, but may blur normative and pathological dissociation (Bernstein and Putnam, 1986). The WERC-DS aims to be more comprehensive, phenomenologically precise and culturally accessible. By integrating established dissociative domains with under-recognized variants such as MD, which assesses features, including yearning, impairment, kinesthetic involvement and music-triggered fantasy immersion, into a single instrument, the WERC-DS supports earlier identification and more accurate differentiation of dissociative symptoms in both research and clinical settings. Nevertheless, the WERC-DS remains an emerging instrument, and additional convergent validity studies comparing it with established measures such as the DES-II are still required.

### Data analysis

Descriptive statistics were used to summarize participant characteristics and item-level responses. Because WERC-DS items are ordinal, polychoric correlation matrices were estimated to examine inter-item associations and to provide the appropriate input for factor analysis.

Exploratory factor analysis was performed to examine the latent structure of the WERC-DS. Eigenvalues and the interpretability of the factor solution guided the number of factors retained. Internal consistency was assessed using Cronbach’s alpha for the total scale and identified subscales. Group differences in dissociative experiences between psychosis high-risk and low-risk psychosis groups were examined using chi-square tests for individual items and independent-samples t-tests for subscale scores. Associations between psychosis, mood and WERC-DS total and subscale scores were examined using correlation analyses. PCA with varimax rotation was selected as an exploratory data-reduction approach because the WERC-DS is still an emerging instrument without an established latent structure in Kenyan or Sub-Saharan African populations. Confirmatory factor analytic approaches were therefore deferred pending further psychometric validation in independent samples. Confirmatory factor analysis was not conducted because the present study represented an initial exploratory examination of the latent structure of the WERC-DS in a Kenyan youth sample. Statistical significance was set at *p* < 0.05.

## Results

### Social demographic characteristics

The study sample comprised 949 participants, with females being the majority at 531 (56.0%), compared to males at 418 (44.0%). Most respondents identified as Protestant at 556 (58.6%), followed by Catholics at 273 (28.8%). A large proportion were never married, 829 (87.4%). Most participants lived in rented houses, 677 (71.5%), and the majority were not employed, 897 (94.8%). The majority were below average (40%) and within the average (22.7) wealth index categories.

### Dissociative experiences by socio-demographics

#### Gender

In the few dissociative experiences that showed significance, males were more likely to report feeling detached from their body (e.g., feeling as if looking at themselves from outside) and noticing changes in colors or sizes of things (item 7: 0.72 ± 1.07 vs. 0.55 ± 1.02, *p* = 0.007). Females, on the other hand, experienced stronger daydreaming tendencies than social engagement others (item 6: 0.91 ± 1.29 vs. 0.61 ± 1.04, *p* < 0.001) (see Supplementary Table 1).

#### Age group

Most items showed no significant differences across age groups. However, a few measures related to daydreaming varied by age significantly. Participants aged 14–18 and 19–24 reported spending more hours daydreaming (item 1: 1.02 ± 0.76 and 1.04 ± 0.90 vs. 0.62 ± 0.72 for 25–30 years, *p* = 0.017) and were more likely to use music or electronics while daydreaming (item 4: 1.23 ± 1.36 and 0.93 ± 1.25 vs. 0.51 ± 1.04, *p* = 0.005). Younger participants also tended to move around, use gestures or make facial expressions while daydreaming (item 5: 0.95 ± 1.29 and 0.82 ± 1.22 vs. 0.46 ± 0.84, *p* = 0.103) and showed a stronger inclination toward daydreaming than spending time with others (item 6: 1.00 ± 1.26 and 0.75 ± 1.19 vs. 0.57 ± 1.04, *p* = 0.060), though these latter differences were marginal (see Supplementary Table 1).

#### Residential context

Most experiences were similar across rural, peri-urban and urban participants. Peri-urban participants reported slightly higher levels on some daydreaming, perceptual and memory-related items compared to those from other groups. They were more prone to daydreaming than to engaging in social interaction (0.84 ± 1.25 vs. 0.66 ± 1.01 rural, 0.60 ± 1.12 Urban, *p* = 0.042), noticed unusual time (1.18 ± 1.26, *p* = 0.003), colors or sizes (0.32 ± 0.73, *p* = 0.009), sounds (0.35 ± 0.77, *p* = 0.022) and memory disruptions (0.48 ± 0.86, *p* = 0.034) (see Supplementary Table 1).

#### Marital status

Difficulty working or completing tasks while daydreaming differed significantly across marital status (*F* = 3.35, *p* = 0.035), with separated (1.60 ± 1.52) and divorced participants (2.67 ± 1.15) reporting higher interference compared to never married (0.97 ± 1.18), in a relationship (0.91 ± 1.27) and married/cohabiting (0.71 ± 1.05) participants. Daydreaming’s interference with daily life also varied significantly (*F* = 4.87, *p* = 0.001), with separated (1.00 ± 1.00) and divorced participants (2.00 ± 1.00) scoring higher than other groups (see Supplementary Table 2).

#### Wealth index

Higher wealth, reflected in participants who were working part-time or volunteering (means ranging from 0.38 to 1.50, SD 0.75–6.60), was more strongly associated with altered body perception (Item 8: 1.50 ± 6.60, *p* < 0.001), unfamiliarity with one’s reflection (Item 9: 0.69 ± 1.09, *p* < 0.001), identity confusion (Item 11: 1.35 ± 1.52, *p* = 0.004) and memory lapses (Item 19: 0.38 ± 0.75, *p* < 0.001), indicating that participants with higher wealth index scores reported higher levels of these dissociative experiences. This finding may reflect contextual factors, such as urban stress, academic pressure and psychosocial burden among higher socioeconomic-status youth, particularly within university and peri-urban environments. Full item-level statistics and group comparisons for marital status and wealth index are presented in Supplementary Table 2.

#### Education level

University students reported higher levels of dissociative experiences on several items compared to tertiary college students. Significant differences included feeling outside their body (item 7: 0.85 ± 1.14 vs. 0.55 ± 1.00, *p* < 0.001), perceiving limbs as unfamiliar (item 8: 0.39 ± 2.12 vs. 0.21 ± 0.66, *p* < 0.001), seeing a different reflection in the mirror (item 9: 0.39 ± 0.85 vs. 0.29 ± 0.76, *p* = 0.004), feeling separated from the world (item 13: 0.36 ± 0.83 vs. 0.21 ± 0.64, *p* < 0.001), noticing unusual visual changes such as altered colors or sizes (item 15: 0.36 ± 0.78 vs. 0.25 ± 0.65, *p* < 0.001) and memory-related disruptions like forgetting how they traveled to a place (item 19: 0.37 ± 0.80 vs. 0.23 ± 0.57, *p* < 0.001). For the remaining items, tertiary college and university students had comparable scores without significant differences (see Supplementary Table 3).

#### Across religious groups

The differences were non-significant for all items with almost similar mean scores among protestants, Catholics, Muslims and other participants (e.g., item 1: 1.06 ± 0.89, 1.01 ± 0.88, 0.89 ± 0.83, 0.85 ± 0.88, *p* = 0.218) (see Supplementary Table 3).

#### Employment

Employment status showed minimal differences in daydreaming and dissociative experiences, with significant variation only for feeling disconnected from thoughts or emotions (*F* = 4.85, *p* = 0.018), experiencing multiple selves (*F* = 5.12, *p* = 0.004) and feeling separated from the world (*F* = 4.75, *p* = 0.006), where working participants reported higher mean scores. This links employment to greater intensity of specific dissociative experiences, potentially reflecting higher cognitive load, stress or environmental demands among working participants compared to their non-working or volunteer counterparts. Detailed item-level statistics for education level, religious affiliation and employment status are provided in Supplementary Table 3.

### Distribution of WERCAP dissociation symptoms

Across the 20 items, mean scores ranged from 0.05 to 1.31, with standard deviations ranging from 0.35 to 1.26. Daydreaming items (Items 1–6) showed higher mean values (*M* = 0.78–1.31) compared to perceptual distortion items (Items 7–16; *M* = 0.22–1.09) and memory lapse items (Items 17–20; *M* = 0.05–0.86). Item-level descriptive statistics for all WERCAP dissociation items are presented in Supplementary Table 4. MD emerged as the most common dissociative experience, with about 26.4% of participants reporting prolonged (>10 h) daily daydreaming, 47% describing their daydreams as almost always being very realistic and 32% noting that daydreaming almost always interfered with work or school activities (see Supplementary Table 5). Additionally, 1.7% of participants reported uniformly high endorsement across all six daydreaming items, corresponding to *“often and almost always”* for frequency-based items or *> 4 h per day* for the time-based daydreaming item.

Changes in time perception were also frequently reported as occurring almost always (39.5%). In contrast, symptoms such as feeling detached from one’s body or surroundings, or having identity-related disturbances, were uncommon, with more than 60% reporting no such experiences and only about 1% showing severe identity-related symptoms. Memory-related dissociation was present at moderate levels, as roughly 28% reported forgetting almost always conversations or recent activities. Overall, dissociative experiences in this group were mostly mild and everyday in nature, mainly involving daydreaming rather than severe dissociative problems. These findings suggest that many dissociative experiences reported in this community sample may reflect normative absorptive or imaginative processes rather than clinically severe dissociative pathology (see Supplementary Table 5).

### Polychoric correlations among WERCAP dissociation

The general correlation was moderate to strong, with a positive trend across items. The strongest relationships were observed among mid-scale items, particularly between items 7–10, 8–9, 10–11, 12–13 and 15–16 (all *r* > 0.60; see Supplementary Table 6). Lower but positive correlations were observed primarily among items 17–20 in relation to earlier items, indicating that all items were related but may tap partially distinct symptom domains.

### KMO and Bartlett’s test results for factor analysis

The data were well-suited for factor analysis. The Kaiser–Meyer–Olkin measure of sampling adequacy was 0.913, indicating excellent sampling adequacy. Bartlett’s test of sphericity was also significant (*χ* ^2^ = 5,558.527, df = 190, *p* < 0.001), indicating that the correlation matrix was factorable, as shown in Supplementary Table 7. Together, these findings confirmed that the dataset met the key requirements for proceeding with factor analysis.

### Exploratory factor analysis of dissociative experience items (PCA, varimax rotation)

There was a clear variation in the item loadings across the three extracted components. All retained items met the minimum loading threshold of 0.45. Perceptual Distortion includes nine items with loadings ranging from 0.466 to 0.721. These items reflected experiences such as altered sensory or environmental perception, emotional disconnection, derealization-like feelings, changes in self-perception, distorted time and out-of-body experiences. Daydream Intensity and.

Engagement contained six items with loadings between 0.629 and 0.742. ITEM 1 had the highest loading (0.742), while the lowest but moderate loading value was 0.629 (ITEM 6). These items related to the intensity and engagement in daydreaming, including time spent daydreaming, realism, interference with tasks, external triggers, physical involvement and preference for solitary fantasy. Memory Lapses consisted of five items with loadings ranging from 0.565 to 0.816.The strongest loading was for ITEM 20 (0.816), followed by 0.781 (ITEM 18), 0.654 (ITEM 8), 0.63 (ITEM 17) and 0.565 (ITEM 19). These items described various forms of memory difficulties, including everyday lapses, forgetting important events, dissociative memory disruptions and depersonalization-related memory issues (See Table 1).

**Table 1.** Exploratory factor analysis of dissociative experience items (PCA, Varimax rotation) Table 1. long description.

Factor	Item	Loading	Interpretation
Factor 1: Perceptual distortion	ITEM 10	0.721	Altered sensory experiences
	ITEM 11	0.721	Altered perception of environment
	ITEM 12	0.65	Dissociation from own emotions
	ITEM 13	0.629	Derealization-like experiences
	ITEM 14	0.598	Feeling of separation from surroundings
	ITEM 15	0.557	Altered self-perception
	ITEM 16	0.534	Distorted time perception
	ITEM 7	0.503	Fragmented sense of self
	ITEM 9	0.466	Out-of-body experiences
	ITEM 8	0.654	Depersonalization-related memory issues
Factor 2: Daydream intensity and engagement	ITEM 1	0.742	Time spent daydreaming
	ITEM 2	0.71	Realism of daydreams
	ITEM 3	0.657	Interference with daily tasks
	ITEM 4	0.645	External triggers for daydreaming
	ITEM 5	0.705	Physical engagement in daydreams
	ITEM 6	0.629	Preference for solitary fantasy
Factor 3: Memory lapses	ITEM 18	0.781	Everyday memory lapses
	ITEM 17	0.63	Forgetting significant events
	ITEM 19	0.565	Dissociative lapses
	ITEM 20	0.816	Severe identity/memory disruption

*Note*: Factor loadings were obtained using principal component analysis (PCA) with varimax rotation. Only loadings ≥0.45 were retained for interpretation.

### Reliability statistics of the dissociation scale and its factors

There was a clear variation in both reliability levels and average item scores across the three extracted factors. The overall dissociation scale showed strong internal consistency (*α* = 0.89). Two of the three subscales also performed well. Perceptual Distortion demonstrated good reliability (*α* = 0.85), and Daydream Intensity and Engagement likewise showed good internal consistency (*α* = 0.81). In contrast, the Memory Lapses subscale showed poor reliability (*α* =0.48) (see Table 2). The relatively low reliability may indicate that memory-related dissociative experiences are more heterogeneous and less stable in community youth populations. Findings related to this subscale should therefore be interpreted cautiously, and future validation studies should evaluate whether some items require refinement or exclusion.

**Table 2.** Reliability statistics of the dissociation scale and its factors Table 2. long description.

	Number of items	Cronbach’s Alpha	Interpretation
Dissociation scale	20	0.89	Good reliability
Perceptual distortion	10	0.85	Good reliability
Daydream intensity & engagement	6	0.81	Good reliability
Memory lapses	4	0.48	Poor reliability

*Note*: Cronbach’s alpha values ≥0.70 indicate acceptable internal consistency, while values below 0.60 reflect poor reliability.

### Association between dissociative experiences and risk level for psychotic disorders

High-risk participants (cut-off score of ≥30 WERCAP) reported significantly more dissociative experiences than low-risk participants, particularly for symptoms involving altered perceptions of the self and reality. For example, daydreaming for 4–10 h daily was reported by 11% of high-risk participants compared to only 4% of low-risk participants (*p* < 0.001), highlighting a greater intensity and frequency of immersive daydreaming in the high-risk group. Similarly, experiences of depersonalization, such as feeling as though one is observing oneself from outside the body, were reported by 17–18% of high-risk respondents compared to 3% of low-risk respondents (*p* = 0.001). Altered body perception (hands or legs not feeling like one’s own) and derealization (feeling separated from the world or observing oneself in the mirror as someone else) also occurred more frequently among high-risk participants (15%), with significant chi-square values (0.001) indicating a robust association between these dissociative experiences and elevated schizophrenia risk (see Table 3).

**Table 3.** Association between dissociative experiences and risk for schizophrenia Table 3. long description.

	0 h		<1 h		1–3 h		4–10 h		>10 h		Sig.
	High risk	Low risk	High risk	Low risk	High risk	Low risk	High risk	Low risk	High risk	Low risk	
1. I usually spend hours daily daydreaming about an imaginary life or event	6(9%)	280(32%)	38(59%)	372(43%)	14(22%)	185(21%)	14(11%)	185(4%)	0(0%)	9(1%)	***p* < 0.001**

	No		Rarely		Sometimes		Often		Almost Always		
	High risk	Low risk	High risk	Low risk	High risk	Low risk	High risk	Low risk	High risk	Low risk	Sig.
2. My daydreams feel very realistic, as if they are really happening	10(15%)	340(39%)	9(14%)	143(16%)	27(42%)	279(32%)	11(17%)	71(8%)	8(12%)	49(6%)	***p* < 0.001**
3. When I daydream, it is very hard to work, study or complete tasks	20(31%)	463(53%)	8(12%)	153(17%)	23(35%)	165(19%)	5(8%)	63(7%)	9(14%)	37(4%)	***p* < 0.001**
4. I use music or electronics to daydream	29(45%)	506(57%)	10(15%)	99(11%)	14(22%)	157(18%)	9(14%)	69(8%)	2(3%)	52(6%)	**0.001**
5. I walk around, move my arms or make expressions with my face when I daydream	28(43%)	553(63%)	11(17%)	109(12%)	13(20%)	119(13%)	8(12%)	52(6%)	4(6%)	49(6%)	***p* < 0.001**
6. I prefer daydreaming than spending time with others	39(60%)	564(64%)	5(8%)	98(11%)	11(17%)	129(15%)	4(6%)	48(5%)	6(9%)	44(5%)	0.535
7. I feel like I am looking at myself from outside my body	30(46%)	610(69%)	11(17%)	102(12%)	13(20%)	119(13%)	6(17%)	29(3%)	5(18%)	23(10%)	**0.001**
8. When I look at my hands or legs, they do not feel like they belong to me	51(78%)	782(89%)	4(6%)	50(6%)	4(6%)	32(4%)	5(8%)	9(1%)	1(15%)	9(1%)	**0.001**
9. When I see myself in the mirror, it feels like I am looking at someone else	47(72%)	733(83%)	6(9%)	65(7%)	6(9%)	56(6%)	3(5%)	17(2%)	3(5%)	8(1%)	**0.025**
10. My thoughts, feelings or emotions do not feel like they are mine	45(69%)	657(74%)	8(12%)	90(10%)	10(15%)	117(13%)	1(2%)	11(1%)	1(15%)	8(1%)	0.912
11. I feel like I am two or more different people with different personalities	34(52%)	576(65%)	7(11%)	99(11%)	15(23%)	134(15%)	2(3%)	41(5%)	7(11%)	33(4%)	**0.022**
12. People or things around me feel strange, like they are part of a dream, movie or video game	45(70%)	673(76%)	6(9%)	99(11%)	3(5%)	76(9%)	5(8%)	24(3%)	5(8%)	9(1%)	***p* < 0.001**
13. It feels like I am separated from the world around me by a glass screen or transparent cloth	56(86%)	754(86%)	4(6%)	62(7%)	4(6%)	45(5%)	1(2%)	11(1%)	0(0%)	9(1%)	0.924
14. It feels like time is moving much faster or slower than it should	27(42%)	423(48%)	4(6%)	120(14%)	24(37%)	242(27%)	5(8%)	47(5%)	5(8%)	50(6%)	0.292
15. The colors or sizes or things around me seem different than they should be	49(75%)	734(83%)	9(14%)	76(9%)	4(6%)	59(7%)	1(2%)	9(1%)	2(3%)	4(1%)	0.06
16. The sounds of people or things seem different than they should be	48(74%)	718(81%)	10(15%)	85(10%)	3(5%)	59(7%)	3(5%)	16(2%)	1(15%)	4(0%)	0.163
17. I forget important or traumatic events that happened to me	37(57%)	547(62%)	11(17%)	156(18%)	10(15%)	116(13%)	4(6%)	41(5%)	3(5%)	22(2%)	0.768
18. I forget conversations I had or things that I did	37(57%)	453(51%)	10(15%)	178(20%)	14(22%)	188(21%)	4(6%)	40(5%)	0(0%)	24(3%)	0.528
19. I forget how I traveled and arrived somewhere	47(72%)	733(83%)	13(20%)	91(10%)	4(6%)	44(5%)	1(2%)	13(1%)	0(0%)	2(0%)	0.175
20. I forget my name or who I am	61(94%)	860(97%)	1(2%)	15(2%)	2(3%)	0(0%)	1(2%)	0(0%)	1(15%)	4(12%)	**0.045**

*Note*: Chi-square analysis indicates that high-risk participants report more dissociative experiences, particularly those involving detachment, while other experiences show no significant difference between risk groups.

Bold values indicate statistically significant results (*p* < 0.05).

### Association of dissociation factors with risk level for psychotic disorders

Participants at high risk for developing psychotic disorders reported significantly higher levels of perceptual distortion and daydream intensity compared to those at low risk (*p* = 0.001 and *p* < 0.001, respectively). In contrast, memory lapses did not differ significantly between the high- and low-risk groups (*p* > 0.05) (see Table 4).

**Table 4.** Association of dissociation factors with risk level for schizophrenia Table 4. long description.

	Participant condition	Mean	Std. deviation	T	Sig.
Perceptual distortion	High risk	6.553846	6.265059	2.7886	**0.001**
	Low risk	4.330691	5.291871		
Daydream intensity	High risk	8.353846	4.932136	4.220407	**<0.001**
	Low risk	5.677237	4.969865		
Memory lapses	High risk	2.6	2.16362	1.827992	0.71
	Low risk	2.08371	2.617522		

*Note*: Means represent average scores for each dissociation factor, with higher values indicating greater levels of dissociative experiences. Statistically significant differences between risk groups are indicated by *p <* 0.05.

### Correlations between psychotic experience severity, dissociation and dissociation subscales

Psychotic experience severity showed no meaningful association with overall dissociation or any of its subscales. Correlations with perceptual distortion (*r* = −0.005), daydream intensity (*r* = −0.004), memory lapses (*r* = 0.007) and total dissociation (*r* = 0.001) were all near zero and non-significant (see Table 5).

**Table 5.** Pearson correlation Table 5. long description.

	Psychosis severity	Perceptual distortion	Daydream intensity	Memory lapses	Dissociation scores
Psychosis severity	1	−0.00526	−0.00394	0.006625	0.001325
Perceptual distortion	−0.00526	1	0.618*	0.444*	0.845*
Daydream intensity	−0.00394	0.618*	1	0.331*	0.891*
Memory lapses	0.006625	0.444*	0.331*	1	0.583*
Dissociation scores	0.001325	0.845*	0.891*	0.583*	1

* Correlation is significant at the 0.01 level (two-tailed).

### Association between bipolar (a-WERCAP) and dissociative experiences

The results show no significant association between dissociative experiences and mood disorder risk (a-WERCAP scores) among youth, as shown in Supplementary Table 8. Mean scores were similar across all frequencies of dissociative experiences, indicating that higher or more frequent dissociation was not associated with increased affective symptoms in this sample.

### Association between dissociative experiences factors and affective symptoms (a-WERCAP)

The dissociation factors, perceptual distortion, daydream intensity and memory lapses were strongly correlated with each other, indicating that these aspects of dissociation tend to co-occur. There was no meaningful correlation between any dissociation factor and affective symptoms (a-WERCAP scores), with all correlations close to zero, as shown in Supplementary Table 9.

## Discussion

The present study provides population-based examinations of dissociative experiences among diverse youth in Africa, focusing on perceptual distortions, daydreaming intensity and memory lapses, and their associations with sociodemographic factors and risk for psychotic and mood disorders. It is also among the first studies internationally to provide detailed data on dissociative symptoms and their relationships with psychosis and mood disorders. Overall, our findings confirm that dissociation is a multidimensional concept that varies across demographic groups and demonstrates meaningful associations with psychiatric vulnerability, aligning with the emerging global literature. Because of the originality of our data, we present all the diverse data by using Supplementary Tables for purposes of comparison in future studies.

### Patterns of dissociative experiences

Immersive daydreaming recorded the highest mean scores across the sample (14–30 years) as compared to perceptual distortions and memory lapses. These patterns were consistent with international findings that absorptive fantasy and immersive daydreaming are common dissociative expressions in youth and non-clinical populations (Soffer-Dudek and Somer, 2022; Sándor et al., 2023). The prominence of immersive daydreaming resonates with the growing MD literature, which identifies immersive fantasy as a distinct dissociative tendency that often emerges during adolescence and young adulthood. Similarly, as noted in an Israeli epidemiological study, MD is particularly common among students and younger adults (Soffer-Dudek and Somer, 2022), mirroring the developmental pattern observed in our sample. A small proportion of participants (1.7%) endorsed the highest response categories (“often” and “almost always”) across immersive daydreaming items, as well as frequent or prolonged engagement (>4 h). Although rare, such extreme patterns may signal a shift from normative absorption toward potentially maladaptive dissociative experiences.

Moreover, the co-occurrence of immersive daydreaming with perceptual and memory-related dissociative phenomena strengthens the position that these experiences represent interrelated dissociative dimensions, rather than manifestations of attentional or affective dysregulation alone. This agrees with the cross-cultural validation studies (Soffer-Dudek et al., 2021) and Hungarian data demonstrating dissociative clustering in identity, imaginative absorption, confusion and impaired control over mental states (Sándor et al., 2023).

### Sociodemographic correlates

Dissociative experiences varied selectively across demographic groups. Gender differences were limited, with males reporting more perceptual-somatic disruptions (e.g., Item 7: 0.72 ± 1.07 vs. 0.55 ± 1.02, *p* = 0.007) and females showing stronger immersive daydreaming (e.g., Item 6: 0.91 ± 1.29 vs. 0.61 ± 1.04, *p* < 0.001). This is in contrast to some Western research, which has found that females have higher levels of dissociation that are usually mediated by trauma exposure or internalizing symptoms (Kate et al., 2020). The limited gender differences in this study may reflect culturally moderated emotional socialization, differential trauma exposure patterns, or reporting differences. Similar null findings have been observed in community-based studies where dissociation is not limited to clinical trauma populations (Ross et al., 1990).

Age differences were statistically significant, with younger participants reporting higher dissociation levels, particularly on daydreaming-related items (for example, Item 1, *p* = 0.017; Item 4, *p* = 0.005), consistent with developmental models of adolescent absorption. This agrees with developmental work showing that dissociative tendencies often peak in adolescence and early adulthood, when identity integration, attachment-related processes and imaginative absorption are heightened (Van IJzendoorn and Schuengel, 1996; Lewis-Fernández and Kirmayer, 2019; Pinto, 2022). This also agrees with MD prevalence patterns from the Israeli epidemiological study, where higher rates were observed among students and young adults (Soffer-Dudek and Somer, 2022). The age gradient observed here, therefore, reinforces the need to consider dissociation as a developmental phenomenon rather than purely a clinical outcome.

Wealth index was significantly associated with dissociation, with high-wealth participants reporting higher levels on several perceptual and identity-related items (e.g., Item 8, *p* < 0.001; Item 9, *p* < 0.001; Item 11, *p* = 0.004; Item 19, *p* < 0.001). This finding contrasts with studies suggesting that socioeconomic disadvantage and exposure to long-term stress and trauma increase the risk of dissociative symptoms (Klest, 2012; Boyer et al., 2022). In the present study, the association may reflect contextual factors, such as urban stress, academic pressures and psychosocial burden among higher socioeconomic-status youth, particularly within university and peri-urban environments. These findings suggest that dissociative experiences in this context may not be exclusively linked to poverty-related adversity but may also emerge within high-pressure social and educational settings among African youth.

### Association between dissociative experiences and psychotic disorder risk (p-WERCAP)

Our results showed that high psychosis risk participants reported significantly more dissociative experiences than low-risk participants, particularly involving altered perceptions of the self and environment (*p* < 0.001). These included depersonalization-like sensations, derealization and prolonged immersive daydreaming.

Although dimensional psychotic experience severity did not correlate with dissociation subscales, categorical high-risk status revealed robust associations. This difference indicates that dissociation may reflect vulnerability rather than symptom severity in non-clinical populations. Dissociative disorders, affecting normally integrated functions, are often underdiagnosed due to clinical overlap with psychotic disorders, despite distinct etiological pathways and care trajectories (Devillé et al., 2014). Moreover, dissociation has been associated with interrupted self-processing, disturbed reality monitoring and disturbed perceptual integration- mechanisms that are also involved in psychosis-spectrum phenomena (Lewis-Fernández and Kirmayer, 2019). One possible explanation is that dissociative experiences may operate through threshold or non-linear relationships, whereby dissociation differentiates broad psychosis-risk categories but does not increase proportionally with symptom severity scores within groups. In addition, the WERCAP severity scale may capture heterogeneous psychotic-like experiences that are not uniformly related to dissociative phenomena.

These findings agree with other studies showing that dissociation can precede or accompany psychotic-like experiences, specifically among traumatized and high-risk individuals (Şar, 2020; Sándor et al., 2023). Epidemiological work on MD has similarly emphasized its dissociative nature and potential diagnostic relevance within dissociative or psychosis-adjacent frameworks (Soffer-Dudek et al., 2021; Soffer-Dudek and Somer, 2022). All these findings confirm the hypothesis that dissociation could be an early vulnerability indication in a youthful population. Dissociative experiences and psychotic symptoms share several phenomenological features, suggesting that they may not represent entirely distinct conditions but rather exist along a continuum of altered subjective experiences (Longden et al., 2020). Experiences such as perceptual distortions, depersonalization and derealization may resemble psychotic-like phenomena, which may partly account for the association between dissociative experiences and psychosis-risk status observed in this study. However, important differences remain, particularly regarding the degree of reality testing and conviction associated with these experiences. Put in a different way, and although at the moment quite a stretch, it is conceivable that the dissociative symptoms and psychotic experiences have a relationship that is modeled after schizoaffective disorders. Further research is needed.

Further to our findings, there was an association between dissociative experiences and psychotic disorder risk. Our analyses showed that 11 dissociative symptoms (Table 3) were significantly associated with high-risk status, predominantly those reflecting derealization, depersonalization and immersive daydreaming. High-risk participants more frequently reported prolonged, realistic and functionally disruptive daydreaming, as well as altered self-perception and perceptual detachment. Consistent with this pattern, factor-level analyses demonstrated that daydream intensity was the most strongly associated dissociation domain, followed by perceptual distortion, both significantly elevated among high-risk individuals (Table 4). In contrast, memory lapses did not significantly differ between groups. All these results suggest that dissociative phenomena related to altered consciousness and self-experience, rather than general forgetfulness, may represent early vulnerability markers for psychotic disorder risk in youth populations.

### Association between dissociative experiences and mood disorder risk (a-WERCAP)

No significant relationship was observed between dissociative experiences and mood disorder risk as measured by the a-WERCAP (all *p* > 0.05). Dissociation frequencies and factor scores were similar across affective symptom categories, and correlations between dissociation subscales and a-WERCAP scores approached zero. This indicates that dissociation in Kenyan youth does not track with affective instability or bipolar symptomatology. This contrasts with some clinical studies reporting dissociation in bipolar patients, often mediated through trauma exposure or rapid cycling (Şar, 2020; Korkmaz, 2024). However, consistent with research asserting that dissociation can constitute an independent psychological dimension rather than an affective disturbance (Pfefferbaum et al., 2006; Krause-Utz and Krause-Utz, 2025). The present findings support a conceptual distinction between dissociation and bipolarity in non-clinical youth.

### Cultural contextualization

Generally, our findings also raise important clinical and public health questions. (1) Are dissociative symptoms predictive of psychotic disorders, and if so, could they be used as an easy-to-administer community screening tool preceding more comprehensive assessments such as the p-WERCAP? (2) Provided dissociative symptom screening could identify youth who were truly at higher risk, it would allow scalable early detection measures in schools, universities and communities, and then provide those who screened positive with WERCAP. This would contribute to the meaningful development of early intervention and prevention paradigms of low-resource areas. On the other hand, it is also important to consider whether the dissociative symptoms are a separate diagnostic entity that is comorbid with psychotic disorders and not just a risk factor. These possibilities are not distinguished by the current findings, though it gives a basis on which longitudinal and clinical validation studies can be conducted in order to answer these pivotal questions within the Kenyan context.

Results from Kenya also align with African dissociation research, indicating that dissociative phenomena are shaped by cultural explanatory systems. Ugandan studies on spirit possession (Braitmayer, 2014) and traditional healing practices (Tobin, 2019) demonstrate different meaning-making systems that can make dissociative states normal or spiritual contexts instead of pathological. On the same note, a post-disaster study done by Pfefferbaum et al. (2006) demonstrated that dissociation can be presented as a culturally normal response of coping. These cultural considerations are essential for interpreting the developmental function and subjective meaning of dissociation in Kenyan youth.

### Limitations and mitigation

1. Self-report measures may be influenced by recall biases or culturally mediated symptom reporting. This limitation can be mitigated by a two-step screening – an initial self-report that may give higher prevalences, followed by researcher-administered screening on those who screen positive on the initial self-report. Those who screen positive on researcher-administered screening can then be referred for further clinical evaluation and follow-up.
2. The wealth index is proxy-based. However, it has been used extensively in past studies.
3. Trauma exposure was not systematically measured, representing a major limitation because trauma is strongly linked to dissociation in existing literature. Consequently, trauma-based explanations discussed in this article remain speculative and should be interpreted cautiously until validated in longitudinal trauma-informed studies.

Future studies ought to embrace longitudinal designs, trauma and attachment measures, as well as culturally sensitive qualitative methodologies of meaning-making regarding dissociation. Confirmatory factor analysis is recommended for future research, ideally with larger, independent samples, to test the stability and validity of the factor solution identified in this study. Neurobiological work may also clarify perceptual integration mechanisms noted in electrophysiological studies.

## Conclusion

This study provides foundational evidence derived from a large-scale community-based screening that dissociative experiences are common, multidimensional and developmentally relevant among Kenyan youth. Dissociation differs by age and socioeconomic status and shows a strong association with psychosis risk symptoms but not with mood disorder risk symptoms. These findings underscore the potential importance of dissociative experiences in the early trajectory of psychotic disorders. Dissociative symptoms may represent phenomenological correlates associated with psychosis-risk experiences and may also constitute a distinct but frequently comorbid dimension within psychotic disorders. If dissociative experiences indeed precede the onset of clinically manifest psychosis, they could serve as a valuable target for early identification and risk stratification, particularly in large-scale community-based screening efforts before the emergence of frank psychotic symptoms. However, the cross-sectional nature of the present findings precludes causal inference. Longitudinal studies are needed to clarify the temporal relationship between dissociation and psychosis risk, to determine whether dissociative symptoms predict transition to psychotic disorders and to establish their utility for early detection and preventive intervention. Future longitudinal and clinically validated studies are necessary before dissociative experiences can be interpreted as predictive markers of psychotic disorders. However, findings should be generalized cautiously because participants were predominantly students from urban and peri-urban settings and may not reflect experiences among rural or non-student youth populations.

## Supporting information

10.1017/gmh.2026.10265.sm001 Ndetei et al. supplementary material Ndetei et al. supplementary material

## Data Availability

Requests for the data may be sent to the corresponding author.

## Supplementary material

The supplementary material for this article can be found at http://doi.org/10.1017/gmh.2026.10265.

## Long descriptions

### Table 1. Long description

The table consists of four columns: Factor, Item, Loading, and Interpretation.

Factor 1: Perceptual distortion includes 10 items:

* ITEM 10: Loading 0.721, Altered sensory experiences.

* ITEM 11: Loading 0.721, Altered perception of environment.

* ITEM 12: Loading 0.65, Dissociation from own emotions.

* ITEM 13: Loading 0.629, Derealization-like experiences.

* ITEM 14: Loading 0.598, Feeling of separation from surroundings.

* ITEM 15: Loading 0.557, Altered self-perception.

* ITEM 16: Loading 0.534, Distorted time perception.

* ITEM 7: Loading 0.503, Fragmented sense of self.

* ITEM 9: Loading 0.466, Out-of-body experiences.

* ITEM 8: Loading 0.654, Depersonalization-related memory issues.

Factor 2: Daydream intensity and engagement includes 6 items:

* ITEM 1: Loading 0.742, Time spent daydreaming.

* ITEM 2: Loading 0.71, Realism of daydreams.

* ITEM 3: Loading 0.657, Interference with daily tasks.

* ITEM 4: Loading 0.645, External triggers for daydreaming.

* ITEM 5: Loading 0.705, Physical engagement in daydreams.

* ITEM 6: Loading 0.629, Preference for solitary fantasy.

Factor 3: Memory lapses includes 4 items:

* ITEM 18: Loading 0.781, Everyday memory lapses.

* ITEM 17: Loading 0.63, Forgetting significant events.

* ITEM 19: Loading 0.565, Dissociative lapses.

* ITEM 20: Loading 0.816, Severe identity/memory disruption.

Note: Factor loadings were obtained using principal component analysis P C A with varimax rotation. Only loadings greater than or equal to 0.45 were retained.

Navigate back to Table 1..

### Table 2. Long description

The table consists of four columns: Factor, Number of items, Cronbach’s Alpha, and Interpretation.

* Dissociation scale: 20 items, Cronbach’s Alpha 0.89, Good reliability.

* Perceptual distortion: 10 items, Cronbach’s Alpha 0.85, Good reliability.

* Daydream intensity and engagement: 6 items, Cronbach’s Alpha 0.81, Good reliability.

* Memory lapses: 4 items, Cronbach’s Alpha 0.48, Poor reliability.

A note below the table specifies that Cronbach’s alpha values greater than or equal to 0.70 indicate acceptable internal consistency, while values below 0.60 reflect poor reliability.

Navigate back to Table 2..

### Table 3. Long description

The table compares High risk and Low risk groups across five frequency categories: 0 h, less than 1 h, 1 to 3 h, 4 to 10 h, and greater than 10 h, with a final column for significance (Sig.).

Key findings include:

* Item 1: Spending hours daydreaming. High risk: 59 percent at less than 1 h. Low risk: 43 percent at less than 1 h. p less than 0.001.

* Item 2: Realistic daydreams. High risk: 42 percent at 1 to 3 h. Low risk: 39 percent at 0 h. p less than 0.001.

* Item 3: Hard to work while daydreaming. High risk: 35 percent at 1 to 3 h. Low risk: 53 percent at 0 h. p less than 0.001.

* Item 5: Physical movements during daydreaming. High risk: 43 percent at 0 h. Low risk: 63 percent at 0 h. p less than 0.001.

* Item 7: Looking at self from outside body. High risk: 46 percent at 0 h. Low risk: 69 percent at 0 h. p equals 0.001.

* Item 12: Surroundings feel strange/dreamlike. High risk: 70 percent at 0 h. Low risk: 76 percent at 0 h. p less than 0.001.

* Item 20: Forgetting name or identity. High risk: 94 percent at 0 h. Low risk: 97 percent at 0 h. p equals 0.045.

Items 6, 10, 13, 14, 15, 16, 17, 18, and 19 show no significant difference between risk groups (p values ranging from 0.06 to 0.924). The data indicates that high-risk participants report more frequent dissociative experiences related to detachment and daydreaming intensity.

Navigate back to Table 3..

### Table 4. Long description

The table contains six columns: Dissociation Factor, Participant condition, Mean, Std. deviation, T, and Sig. (significance).

* Perceptual distortion: High risk group has a Mean of 6.553846 and Std. deviation of 6.265059. Low risk group has a Mean of 4.330691 and Std. deviation of 5.291871. The T value is 2.7886 with a Sig. of 0.001.

* Daydream intensity: High risk group has a Mean of 8.353846 and Std. deviation of 4.932136. Low risk group has a Mean of 5.677237 and Std. deviation of 4.969865. The T value is 4.220407 with a Sig. of less than 0.001.

* Memory lapses: High risk group has a Mean of 2.6 and Std. deviation of 2.16362. Low risk group has a Mean of 2.08371 and Std. deviation of 2.617522. The T value is 1.827992 with a Sig. of 0.71.

Note: Higher mean values indicate greater levels of dissociative experiences. Statistically significant differences (p less than 0.05) are observed for Perceptual distortion and Daydream intensity.

Navigate back to Table 4..

### Table 5. Long description

The table is a 5 by 5 correlation matrix. The diagonal values are all 1, representing the correlation of each variable with itself.

* Psychosis severity shows very weak, non-significant correlations with all other variables: minus 0.00526 with perceptual distortion, minus 0.00394 with daydream intensity, 0.006625 with memory lapses, and 0.001325 with dissociation scores.

* Perceptual distortion has significant positive correlations with daydream intensity (0.618), memory lapses (0.444), and dissociation scores (0.845).

* Daydream intensity has significant positive correlations with memory lapses (0.331) and dissociation scores (0.891).

* Memory lapses has a significant positive correlation with dissociation scores (0.583).

Asterisks indicate that correlations are significant at the 0.01 level (two-tailed). The strongest relationship in the data is between daydream intensity and dissociation scores (0.891).

Navigate back to Table 5..

## References

[r1] Allen JG, Coyne L and Console DA (1997) Dissociative detachment relates to psychotic symptoms and personality decompensation. Comprehensive Psychiatry 38(6), 327–334. 10.1016/S0010-440X(97)90928-7. 9406738

[r2] Bernstein EM and Putnam FW (1986) Development, reliability, and validity of a dissociation scale. The Journal of Nervous and Mental Disease 174(12), 727–735. 3783140 10.1097/00005053-198612000-00004

[r3] Boyer SM, Caplan JE and Edwards LK (2022) Trauma-related dissociation and the dissociative disorders: Neglected symptoms with severe public health consequences. Delaware Journal of Public Health 8(2), 78–84. 10.32481/djph.2022.05.010. PMC9162402 35692991

[r4] Braitmayer L (2014) Ghosts of the past. A critical review of the relationship between trauma exposure, spiritual possession and dissociative disorders. European Journal of Psychotraumatology 6(10), 3402.

[r5] Cernis EA (2020) Dissociation in Non-affective Psychosis: A Felt Sense of Anomaly. Oxford, UK: University of Oxford.

[r6] Dell PF (2009) The phenomena of pathological dissociation. In Dell PF and O’Neil JA (eds), Dissociation and the Dissociative Disorders: DSM-V and beyond. New York: Routledge, pp. 225–237.

[r7] Dell PF and O’Neil JA (2010) Dissociation and the Dissociative Disorders: DSM-V and beyond. New York: Routledge. 10.4324/9780203893920.

[r8] Devillé C, Moeglin C and Sentissi O (2014) Dissociative disorders: Between neurosis and psychosis. Case Reports in Psychiatry 2014(1), 1–6. 10.1155/2014/425892. PMC4227384 25405051

[r9] Ferrajao P, Tourais B and Elklit A (2024) Attachment anxiety and dissociation mediate associations between polytrauma and somatization in Kenyan adolescents. Journal of Trauma & Dissociation 25(1), 83–98. 10.1080/15299732.2023.2231958. 37401367

[r10] Kate M-A, Hopwood T and Jamieson G (2020) The prevalence of dissociative disorders and dissociative experiences in college populations: A meta-analysis of 98 studies. Journal of Trauma & Dissociation 21(1), 16–61. 10.1080/15299732.2019.1647915. 31461395

[r11] Klest B (2012) Childhood trauma, poverty, and adult victimization. Psychological Trauma: Theory, Research, Practice, and Policy 4(3), 245–251. 10.1037/a0024468.

[r12] Kohl KL (2010) Trauma, Dissociation, and Traumatic Stress at a Trauma Center Serving Low-Income Children and Adolescents. Chicago, IL: Loyola University Chicago.

[r13] Korkmaz Ş (2024) Important research of the past year in the field of mood disorders: Studies on treatment options in bipolar disorder. Psychiatry and Clinical Psychopharmacology 35, 27.

[r14] Krause-Utz A and Krause-Utz A (2025) Mental disorders associated with dissociation. In Dissociation in Borderline Personality Disorder: Associations with Trauma and Neurobiology. Cham: Springer, pp. 9–29. 10.1007/978-3-032-06339-72.

[r15] Lewis-Fernández R and Kirmayer LJ (2019) Cultural concepts of distress and psychiatric disorders: Understanding symptom experience and expression in context. Transcultural Psychiatry 56(4), 786–803. 10.1177/1363461519861795. 31347476

[r16] Longden E, Branitsky A, Moskowitz A, Berry K, Bucci S and Varese F (2020) The relationship between dissociation and symptoms of psychosis: A meta-analysis. Schizophrenia Bulletin 46(5), 1104–1113. 10.1093/schbul/sbaa037. 32251520 PMC7505175

[r17] Lyssenko L, Schmahl C, Bockhacker L, Vonderlin R, Bohus M and Kleindienst N (2018) Dissociation in psychiatric disorders: A meta-analysis of studies using the dissociative experiences scale. American Journal of Psychiatry 175(1), 37–46. 10.1176/appi.ajp.2017.17010025. 28946763

[r18] Mamah D (2011) The Washington Early Recognition Center Affectivity and Psychosis (WERCAP) Screen. Missouri: Washington University, St. Louis. 10.1016/j.psychres.2019.112569 31727439

[r19] Moskowitz A, Schäfer I and Dorahy MJ (2011) Psychosis, Trauma and Dissociation: Emerging Perspectives on Severe Psychopathology. Hoboken, NJ, USA: Wiley-Blackwell.

[r20] Murphy RJ (2023) Depersonalization/derealization disorder and neural correlates of trauma-related pathology: A critical review. Innovations in Clinical Neuroscience 20(1–3), 53–59. PMC10132272 37122581

[r21] Ndetei D, Pike K, Mutiso V, Tele A, Gitonga I, Rebello T, Musyimi C and Mamah D (2019) The psychometric properties of the Washington Early Recognition Center Affectivity and Psychosis (WERCAP) screen in adults in the Kenyan context: Towards combined large scale community screening for affectivity and psychosis. Psychiatry Research 282, 112569. 10.1016/j.psychres.2019.112569. 31727439

[r22] Pfefferbaum B, North CS, Doughty DE, Pfefferbaum RL, Dumont CE, Pynoos RS, Gurwitch RH and Ndetei D (2006) Trauma, grief and depression in Nairobi children after the 1998 bombing of the American embassy. Death Studies 30(6), 561–577. 10.1080/07481180600742566. 16773776

[r23] Pienkos E, Giersch A, Hansen M, Humpston C, McCarthy-Jones S, Mishara A, Nelson B, Park S, Raballo A, Sharma R, Thomas N and Rosen C (2019) Hallucinations beyond voices: A conceptual review of the phenomenology of altered perception in psychosis. Schizophrenia Bulletin 45(Supplement_1), S67–S77. 10.1093/schbul/sby057. 30715544 PMC6357976

[r24] Pini S, Nardi B, Carpita B, Lorenzi G, Mula M, Milrod B, Massimetti G, Cremone IM, Bonelli C, Domschke K, Schiele M, Dell’Osso L and Baldwin DS (2025) Relationships between depersonalization-derealization symptoms and separation anxiety in adult patients with mood and anxiety disorders. Frontiers in Psychiatry 16, 1565217. 10.3389/fpsyt.2025.1565217. 40405883 PMC12095167

[r25] Pinto, B. P. G. T. (2022). *Attachment and dissociation mediate associations between polyvictimization and somatization differently in Kenyan male and female adolescents.*

[r26] Ross CA, Joshi S and Currie R (1990) Dissociative experiences in the general population. American Journal of Psychiatry 147(11), 1547–1552. 10.1176/ajp.147.11.1547. 2221172

[r27] Saini N, Bhowmick I and Kumar P (2024) Socio-demographics determinants, clinical correlates and family environment among dissociative disorder. International Journal of Indian Psychology 12, 277–286.

[r28] Sándor A, Bugán A, Nagy A, Nagy N, Tóth-Merza K and Molnár J (2023) Childhood traumatization and dissociative experiences among maladaptive and normal daydreamers in a Hungarian sample. Current Psychology 42(11), 9509–9525. 10.1007/s12144-021-02223-3. 34483632 PMC8403514

[r29] Şar V (2020) Childhood trauma and dissociative disorders. In Childhood Trauma in Mental Disorders: A Comprehensive Approach. Cham: Springer, pp. 333–365. 10.1007/978-3-030-49414-8_16.

[r30] Schiavone FL, McKinnon MC and Lanius RA (2018) Psychotic-like symptoms and the temporal lobe in trauma-related disorders: Diagnosis, treatment, and assessment of potential malingering. Chronic Stress 2, 2470547018797046. 10.1177/2470547018797046. 32440584 PMC7219949

[r31] Soffer-Dudek N and Somer E (2022) Maladaptive daydreaming is a dissociative disorder. In Maladaptive Daydreaming Is a Dissociative Disorder. New York: Routledge, pp. 547–563. 10.4324/9781003057314-41.

[r32] Soffer-Dudek N, Somer E, Abu-Rayya HM, Metin B and Schimmenti A (2021) Different cultures, similar daydream addiction? An examination of the cross-cultural measurement equivalence of the maladaptive daydreaming scale. Journal of Behavioral Addictions 9(4), 1056–1067. 10.1556/2006.2020.00080. PMC8969720 33141115

[r33] Soffer-Dudek N, Somer E, Spiegel D, Chefetz R, O’Neil J, Dorahy MJ, Cardeña E, Mamah D, Schimmenti A, Musetti A, Boon S, van Dijke A, Ross C, Nijenhuis E, Krause-Utz A, Dell P, Gold SN, Pietkiewicz I, Silberg J, Steele K, Moskowitz A, Draijer N, Thomson P, Barach P, Kinsler P, Maves P, Şar V, Krüger C and Middleton W (2025) Maladaptive daydreaming should be included as a dissociative disorder in psychiatric manuals: Position paper. The British Journal of Psychiatry 226(4), 238–242. 10.1192/bjp.2024.279. 40094484 PMC12038384

[r34] Somer E, Lehrfeld J, Bigelsen J and Jopp DS (2016) Development and validation of the maladaptive daydreaming scale (MDS). Consciousness and Cognition 39, 77–91. 10.1016/j.concog.2015.12.001. 26707384

[r35] Somer E, Soffer-Dudek N, Ross CA and Halpern N (2017) Maladaptive daydreaming: Proposed diagnostic criteria and their assessment with a structured clinical interview. Psychology of Consciousness: Theory, Research, and Practice 4(2), 176–189. 10.1037/cns0000114.

[r36] Spiegel D, Loewenstein RJ, Lewis-Fernández R, Sar V, Simeon D, Vermetten E, Cardeña E and Dell PF (2011) Dissociative disorders in DSM-5. Depression and Anxiety 28(9), 824–852. 10.1002/da.20874. 21910187

[r37] Tobin SM (2019) Exorcism, Deliverance, and Psychotherapy from a Catholic-Christian Perspective: A Critical Literature Review. Azusa Pacific University.

[r38] Van IJzendoorn MH and Schuengel C (1996) The measurement of dissociation in normal and clinical populations: Meta-analytic validation of the dissociative experiences scale (DES). Clinical Psychology Review 16(5), 365–382. 10.1016/0272-7358(96)00006-2.

[r39] Van Os J, Linscott RJ, Myin-Germeys I, Delespaul P and Krabbendam L (2009) A systematic review and meta-analysis of the psychosis continuum: Evidence for a psychosis proneness–persistence–impairment model of psychotic disorder. Psychological Medicine 39(2), 179–195. 10.1017/S0033291708003814. 18606047

