# Supplementary material for: Dissociative experiences among Kenyan youth in the Nairobi metropolitan area: Associations with psychosis risk symptoms
Source: Glob Ment Health (Camb). 2026 Jun 25;13:e158. doi: 10.1017/gmh.2026.10265 (PMC13433326; doi:10.1017/gmh.2026.10265)
Supplement: Supplementary file 1 [file S2054425126102659sup001.docx]

| **Supplementary Table 1: Dissociation Items versus gender, age group, and residence** | | | | | | | | | | | |
| --- | --- | --- | --- | --- | --- | --- | --- | --- | --- | --- | --- |
|  | **Gender** | | | **Age group** | | | | **Residence** | | | |
|  | **Female** | **Male** |  | **14–18 years** | **19–24 years** | **25–30 years** | | **Rural** | **Peri-Urban** | **Urban** |  |
| **Dissociation Items** | **Mean (Std. Deviation)** | **Mean (Std. Deviation)** | **Sig.** | **Mean (Std. Deviation)** | **Mean (Std. Deviation)** | **Mean (Std. Deviation)** | **Sig.** | **Mean (Std. Deviation)** | **Mean (Std. Deviation)** | **Mean (Std. Deviation)** | **Sig.** |
| 1. I usually spend _____ hours daily daydreaming about an imaginary life or event | 1.00 (0.92) | 1.05 (0.82) | 0.4 | 1.02 (0.76) | 1.04 (0.90) | 0.62 (0.72) | **0.017** | 0.97 (0.82) | 1.04 (0.89) | 0.98 (0.89) | 0.593 |
| 2. My daydreams feel very realistic, as if they are really happening | 1.28 (1.23) | 1.35 (1.21) | 0.37 | 1.41 (1.24) | 1.31 (1.21) | 0.81 (1.15) | **0.031** | 1.29 (1.29) | 1.34 (1.21) | 1.14 (1.17) | 0.214 |
| 3. When I daydream, it is very hard to work, study or complete tasks | 0.98 (1.21) | 0.98 (1.18) | 0.95 | 0.98 (1.08) | 0.99 (1.22) | 0.59 (0.83) | 0.146 | 0.95 (1.17) | 0.99 (1.21) | 0.95 (1.20) | 0.878 |
| 4. I use music or electronics to daydream | 0.92 (1.26) | 0.99 (1.27) | 0.41 | 1.23 (1.36) | 0.93 (1.25) | 0.51 (1.04) | **0.005** | 0.86 (1.17) | 0.98 (1.30) | 0.95 (1.19) | 0.565 |
| 5. I walk around, move my arms or make expressions with my face when I daydream | 0.84 (1.22) | 0.80 (1.23) | 0.65 | 0.95 (1.29) | 0.82 (1.22) | 0.46 (0.84) | 0.101 | 0.70 (1.13) | 0.87 (1.25) | 0.75 (1.18) | 0.236 |
| 6. I prefer daydreaming than spending time with others | 0.91 (1.29) | 0.61 (1.04) | **<0.001** | 1.00 (1.26) | 0.75 (1.19) | 0.57 (1.04) | 0.06 | 0.66 (1.01) | 0.84 (1.25) | 0.60 (1.12) | **0.042** |
| 7. I feel like I am looking at myself from outside my body | 0.55 (1.02) | 0.72 (1.07) | **0.011** | 0.57 (1.02) | 0.64 (1.05) | 0.46 (0.90) | 0.507 | 0.67 (1.08) | 0.62 (1.04) | 0.60 (1.02) | 0.85 |
| 8. When I look at my hands or legs, they don’t feel like they belong to me | 0.22 (0.70) | 0.22 (0.67) | 0.94 | 0.15 (0.55) | 0.23 (0.68) | 0.27 (0.93) | 0.484 | 0.43 (2.56) | 0.22 (0.67) | 0.20 (0.71) | 0.821 |
| 9. When I see myself in the mirror, it feels like I am looking at someone else | 0.33 (0.79) | 0.31 (0.78) | 0.71 | 0.36 (0.86) | 0.31 (0.77) | 0.22 (0.79) | 0.621 | 0.33 (0.81) | 0.34 (0.80) | 0.19 (0.68) | 0.149 |
| 10. My thoughts, feelings or emotions don’t feel like they are mine | 0.48 (0.85) | 0.41 (0.82) | 0.173 | 0.43 (0.72) | 0.45 (0.85) | 0.30 (0.81) | 0.555 | 0.43 (0.87) | 0.48 (0.86) | 0.28 (0.68) | **0.034** |
| 11. I feel like I am two or more different people with different personalities | 0.73 (1.16) | 0.73 (1.10) | 0.962 | 0.90 (1.26) | 0.72 (1.12) | 0.41 (0.93) | 0.056 | 0.67 (1.13) | 0.76 (1.14) | 0.65 (1.11) | 0.449 |
| 12. People or things around me feel strange, like they are part of a dream, movie or video game | 0.45 (0.90) | 0.41 (0.85) | 0.489 | 0.52 (0.97) | 0.42 (0.87) | 0.24 (0.64) | 0.237 | 0.31 (0.76) | 0.48 (0.92) | 0.33 (0.78) | **0.049** |
| 13. It feels like I am separated from the world around me by a glass screen or transparent cloth | 0.25 (0.70) | 0.25 (0.69) | 0.998 | 0.18 (0.59) | 0.25 (0.69) | 0.32 (0.97) | 0.425 | 0.24 (0.65) | 0.27 (0.73) | 0.17 (0.56) | 0.337 |
| 14. It feels like time is moving much faster or slower than it should | 1.07 (1.23) | 1.12 (1.21) | 0.547 | 1.08 (1.20) | 1.10 (1.23) | 1.00 (1.27) | 0.889 | 0.87 (1.12) | 1.18 (1.26) | 0.91 (1.11) | **0.003** |
| 15. The colors or sizes of things around me seem different than they should be | 0.25 (0.64) | 0.32 (0.75) | 0.138 | 0.32 (0.71) | 0.27 (0.67) | 0.35 (0.86) | 0.565 | 0.24 (0.67) | 0.32 (0.73) | 0.12 (0.41) | **0.009** |
| 16. The sounds of people or things seem different than they should be | 0.31 (0.76) | 0.31 (0.69) | 0.976 | 0.31 (0.74) | 0.32 (0.74) | 0.19 (0.46) | 0.586 | 0.28 (0.70) | 0.35 (0.77) | 0.16 (0.50) | **0.022** |
| 17. I forget important or traumatic events that happened to me | 0.68 (1.04) | 0.70 (1.04) | 0.801 | 0.62 (0.98) | 0.71 (1.05) | 0.46 (0.99) | 0.255 | 0.74 (1.05) | 0.71 (1.05) | 0.53 (0.95) | 0.18 |
| 18. I forget conversations I had or things that I did | 0.89 (1.09) | 0.83 (1.02) | 0.365 | 0.92 (1.14) | 0.86 (1.05) | 0.78 (1.03) | 0.738 | 0.74 (1.05) | 0.92 (1.08) | 0.73 (0.99) | 0.052 |
| 19. I forget how I traveled and arrived somewhere | 0.24 (0.62) | 0.29 (0.66) | 0.26 | 0.25 (0.69) | 0.27 (0.64) | 0.16 (0.44) | 0.592 | 0.23 (0.64) | 0.27 (0.65) | 0.25 (0.57) | 0.7 |
| 20. I forget my name or who I am | 0.05 (0.35) | 0.06 (0.36) | 0.726 | 0.09 (0.47) | 0.05 (0.34) | 0.03 (0.16) | 0.36 | 0.06 (0.40) | 0.06 (0.37) | 0.01 (0.09) | 0.316 |
| ***Note:*** *Values are shown as Mean (SD); Sig. represents the p-value, where p < 0.05 is bolded, indicating a statistically significant difference.* | | | | | | | | | | | |

| **Supplementary Table 2: Dissociation items vs marital status and wealth index** | | | | | | | | | |
| --- | --- | --- | --- | --- | --- | --- | --- | --- | --- |
|  | **Marital status** | | | | | | | **Wealth index** | |
| **Dissociation Items** | **Never married** | **In a relationship** | **Married/cohabiting** | **Separated** | **Divorced** | **Widowed** |  |  |  |
|  | **Mean (Std. Deviation)** | **Mean (Std. Deviation)** | **Mean (Std. Deviation)** | **Mean (Std. Deviation)** | **Mean (Std. Deviation)** | **Mean (Std. Deviation)** | **Sig.** | **Pearson Correlation** | **Sig.** |
| 1. I usually spend _____ hours daily daydreaming about an imaginary life or event | 1.02 (0.88) | 1.18 (0.96) | 0.59 (0.62) | 1.40 (0.55) | 1.00 (0.00) | 1.00 (0.00) | 0.287 | -0.024 | 0.46 |
| 2. My daydreams feel very realistic, as if they are really happening | 1.31 (1.21) | 1.73 (1.49) | 1.12 (1.36) | 2.00 (0.00) | 2.00 (1.00) | 0.50 (0.71) | 0.076 | 0.006 | 0.854 |
| 3. When I daydream, it is very hard to work, study or complete tasks | 0.97 (1.18) | 0.91 (1.27) | 0.71 (1.05) | 1.60 (1.52) | 2.67 (1.15) | 2.00 (0.00) | 0.035 | -0.046 | 0.157 |
| 4. I use music or electronics to daydream | 0.93 (1.25) | 1.32 (1.43) | 0.76 (1.25) | 0.60 (0.55) | 0.67 (1.15) | 1.00 (1.41) | 0.112 | .101** | **0.002** |
| 5. I walk around, move my arms or make expressions with my face when I daydream | 0.81 (1.21) | 1.05 (1.62) | 0.82 (1.18) | 0.60 (0.89) | 0.33 (0.58) | 0.00 (0.00) | 0.385 | .096** | **0.003** |
| 6. I prefer daydreaming than spending time with others | 0.77 (1.19) | 1.05 (1.43) | 0.47 (0.72) | 1.60 (1.52) | 1.33 (0.58) | 0.50 (0.71) | 0.744 | .120** | **p<0.001** |
| 7. I feel like I am looking at myself from outside my body | 0.62 (1.03) | 0.64 (1.14) | 0.59 (1.18) | 0.80 (1.10) | 0.67 (0.58) | 0.50 (0.71) | 0.166 | .074* | **0.024** |
| 8. When I look at my hands or legs, they don’t feel like they belong to me | 0.22 (0.68) | 1.50 (6.60) | 0.24 (0.56) | 0.20 (0.45) | 0.00 (0.00) | 0.00 (0.00) | 0.73 | .654** | **p<0.001** |
| 9. My daydreams are often linked with real-life events | 1.19 (1.21) | 1.32 (1.29) | 1.18 (1.15) | 0.60 (0.55) | 1.00 (1.00) | 1.00 (0.00) | 0.65 | .349** | **p<0.001** |
| 10. My daydreams are very detailed and vivid | 1.42 (1.23) | 1.59 (1.47) | 1.29 (1.26) | 1.80 (1.10) | 2.00 (1.00) | 0.50 (0.71) | 0.535 | .141** | **p<0.001** |
| 11. I find it easy to stop daydreaming when I need to focus | 0.94 (1.13) | 0.82 (1.01) | 0.82 (1.15) | 1.00 (1.00) | 1.67 (0.58) | 1.00 (0.00) | 0.139 | .182** | **p<0.001** |
| 12. My daydreams interfere with my normal life or activities | 0.84 (1.13) | 0.95 (1.36) | 0.71 (0.99) | 1.00 (1.00) | 2.00 (1.00) | 1.00 (1.41) | **0.001** | 0.022 | 0.493 |
| 13. My daydreams are about fictional characters, celebrities, or imaginary worlds | 0.82 (1.18) | 1.00 (1.15) | 0.59 (0.80) | 0.80 (0.84) | 0.33 (0.58) | 1.00 (1.41) | 0.426 | .196** | **p<0.001** |
| 14. I sometimes confuse my daydreams with real memories | 0.46 (0.91) | 0.77 (1.25) | 0.53 (1.01) | 0.40 (0.55) | 0.33 (0.58) | 0.00 (0.00) | 0.204 | -.077* | **0.018** |
| 15. I think about my daydreams more than my real life | 0.59 (1.02) | 0.73 (1.19) | 0.53 (0.94) | 1.00 (1.00) | 1.00 (1.00) | 0.00 (0.00) | 0.11 | -0.012 | 0.705 |
| 16. I have trouble sleeping because I stay up daydreaming | 0.36 (0.83) | 0.41 (0.91) | 0.24 (0.66) | 0.60 (0.89) | 1.00 (1.00) | 0.00 (0.00) | 0.18 | -0.023 | 0.49 |
| 17. I have friends or relatives who complain about the time I spend daydreaming | 0.29 (0.75) | 0.36 (0.79) | 0.18 (0.53) | 0.20 (0.45) | 0.00 (0.00) | 0.00 (0.00) | 0.91 | -.068* | **0.038** |
| 18. I prefer to daydream in private | 0.83 (1.17) | 1.14 (1.37) | 0.65 (1.17) | 1.40 (1.14) | 1.67 (0.58) | 1.00 (0.00) | 0.356 | .071* | **0.03** |
| 19. I have tried to cut down on daydreaming but could not | 0.73 (1.10) | 0.95 (1.43) | 0.76 (1.15) | 1.00 (1.00) | 1.33 (0.58) | 0.50 (0.71) | 0.379 | .478** | **p<0.001** |
| 20. Daydreaming helps me cope with loneliness or stress | 1.00 (1.23) | 1.14 (1.23) | 0.88 (1.17) | 1.80 (1.10) | 2.00 (1.00) | 1.50 (0.71) | 0.996 | .816** | **p<0.001** |
| ***Note:*** *Values are shown as Mean (SD); Sig. represents the p-value, where p < 0.05 is bolded, indicating a statistically significant difference.* | | | | | | | | | |

| **Supplementary Table 3: Dissociation items versus education level, religion, and employment status** | | | | | | | | | | | | | |
| --- | --- | --- | --- | --- | --- | --- | --- | --- | --- | --- | --- | --- | --- |
|  | **level of education** | | | **Religion** | | | | | **Employment** | | | | |
|  | **Tertiary College** | **University** |  | **Protestant** | **Catholic** | **Muslim** | **Others** |  | **No** | **Volunteering** | **Full-time** | **Part-time** | |
| **Dissociation Items** | **Mean (SD)** | **Mean (SD)** | **Sig.** | **Mean (SD)** | **Mean (SD)** | **Mean (SD)** | **Mean (SD)** | **Sig.** | **Mean (SD)** | **Mean (SD)** | **Mean (SD)** | **Mean (SD)** | **Sig.** |
| 1. I usually spend _____ hours daily daydreaming about an imaginary life or event | 0.98(0.87) | 1.14(0.90) | 0.092 | 1.06 (0.89) | 1.01 (0.88) | 0.89 (0.83) | 0.85 (0.88) | 0.218 | 1.02 (0.88) | 1.18 (0.96) | 0.00 (0.00) | 1.00 (0.96) | 0.554 |
| 2. My daydreams feel very realistic, as if they are really happening | 1.28(1.22) | 1.40(1.23) | 0.855 | 1.35 (1.23) | 1.26 (1.22) | 1.25 (1.21) | 1.20 (1.08) | 0.796 | 1.31 (1.21) | 1.73 (1.49) | 1.00 (0.00) | 1.00 (1.23) | 0.23 |
| 3. When I daydream, it is very hard to work, study or complete tasks | 0.95(1.19) | 1.05(1.22) | 0.546 | 0.98 (1.22) | 1.01 (1.20) | 0.96 (1.07) | 0.78 (1.24) | 0.746 | 0.97 (1.18) | 0.91 (1.27) | 0.00 (0.00) | 1.23 (1.50) | 0.587 |
| 4. I use music or electronics to daydream | 0.94(1.26) | 1.00(1.29) | 0.556 | 0.96 (1.25) | 0.92 (1.27) | 1.09 (1.43) | 0.83 (1.14) | 0.703 | 0.93 (1.25) | 1.32 (1.43) | 1.00 (0.00) | 1.50 (1.50) | 0.072 |
| 5. I walk around, move my arms or make expressions with my face when I daydream | 0.79(1.21) | 0.94(1.27) | 0.254 | 0.91 (1.28) | 0.77 (1.16) | 0.59 (1.16) | 0.51 (0.95) | **0.045** | 0.81 (1.20) | 1.05 (1.62) | 1.00 (0.00) | 1.27 (1.48) | 0.229 |
| 6. I prefer daydreaming than spending time with others | 0.77(1.20) | 0.80(1.17) | 0.79 | 0.78 (1.20) | 0.78 (1.20) | 0.72 (1.23) | 0.78 (1.15) | 0.874 | 0.77 (1.19) | 1.05 (1.43) | 0.00 (0.00) | 1.08 (1.32) | 0.357 |
| 7. I feel like I am looking at myself from outside my body | 0.55(1.00) | 0.85(1.14) | **P<0.001** | 0.64 (1.02) | 0.56 (1.03) | 0.78 (1.21) | 0.59 (1.16) | 0.467 | 0.62 (1.03) | 0.64 (1.14) | 0.00 (0.00) | 0.92 (1.47) | 0.477 |
| 8. When I look at my hands or legs, they don’t feel like they belong to me | 0.21(0.66) | 0.39(2.12) | **P<0.001** | 0.27 (1.47) | 0.19 (0.65) | 0.41 (0.98) | 0.10 (0.44) | 0.573 | 0.22 (0.68) | 0.23 (0.75) | 0.00 (0.00) | 0.38 (0.75) | **P<0.001** |
| 9. When I see myself in the mirror, it feels like I am looking at someone else | 0.29(0.76) | 0.39(0.85) | **0.004** | 0.32 (0.77) | 0.26 (0.73) | 0.53 (1.07) | 0.29 (0.75) | 0.13 | 0.31 (0.78) | 0.18 (0.66) | 0.00 (0.00) | 0.69 (1.09) | 0.078 |
| 10. My thoughts, feelings or emotions don’t feel like they are mine | 0.42(0.81) | 0.52(0.91) | **0.014** | 0.44 (0.82) | 0.48 (0.88) | 0.44 (0.87) | 0.46 (0.84) | 0.932 | 0.43 (0.81) | 0.64 (1.18) | 0.00 (0.00) | 0.92 (1.32) | **0.018** |
| 11. I feel like I am two or more different people with different personalities | 0.71(1.14) | 0.80(1.13) | 0.927 | 0.76 (1.14) | 0.67 (1.14) | 0.63 (1.03) | 0.88 (1.27) | 0.484 | 0.70 (1.10) | 1.23 (1.63) | 0.00 (0.00) | 1.35 (1.52) | **0.004** |
| 12. People or things around me feel strange, like they are part of a dream, movie or video game | 0.41(0.86) | 0.49(0.95) | 0.057 | 0.43 (0.86) | 0.39 (0.86) | 0.54 (0.98) | 0.51 (1.03) | 0.649 | 0.42 (0.88) | 0.41 (0.80) | 0.00 (0.00) | 0.69 (1.05) | 0.459 |
| 13. It feels like I am separated from the world around me by a glass screen or transparent cloth | 0.21(0.64) | 0.36(0.83) | **P<0.001** | 0.25 (0.72) | 0.23 (0.62) | 0.25 (0.65) | 0.24 (0.66) | 0.988 | 0.23 (0.67) | 0.50 (0.91) | 0.00 (0.00) | 0.65 (1.13) | **0.006** |
| 14. It feels like time is moving much faster or slower than it should | 1.03(1.21) | 1.28(1.27) | 0.105 | 1.09 (1.22) | 1.05 (1.22) | 1.14 (1.24) | 1.29 (1.38) | 0.813 | 1.08 (1.21) | 1.18 (1.44) | 0.00 (0.00) | 1.46 (1.61) | 0.327 |
| 15. The colors or sizes of things around me seem different than they should be | 0.25(0.65) | 0.36(0.78) | **P<0.001** | 0.27 (0.65) | 0.29 (0.71) | 0.32 (0.75) | 0.37 (0.94) | 0.879 | 0.27 (0.68) | 0.23 (0.61) | 0.00 (0.00) | 0.54 (1.07) | 0.262 |
| 16. The sounds of people or things seem different than they should be | 0.30(0.73) | 0.35(0.73) | 0.287 | 0.30 (0.70) | 0.35 (0.80) | 0.29 (0.66) | 0.32 (0.79) | 0.896 | 0.30 (0.70) | 0.45 (0.91) | 0.00 (0.00) | 0.65 (1.23) | 0.073 |
| 17. I forget important or traumatic events that happened to me | 0.67(1.02) | 0.75(1.09) | 0.285 | 0.69 (1.05) | 0.60 (0.94) | 0.78 (1.09) | 0.95 (1.22) | 0.22 | 0.68 (1.03) | 0.64 (1.05) | 0.00 (0.00) | 0.92 (1.35) | 0.611 |
| 18. I forget conversations I had or things that I did | 0.84(1.04) | 0.94(1.13) | 0.352 | 0.87 (1.08) | 0.81 (1.01) | 0.81 (1.08) | 1.20 (0.98) | 0.227 | 0.86 (1.04) | 0.68 (1.09) | 0.00 (0.00) | 1.31 (1.49) | 0.118 |
| 19. I forget how I traveled and arrived somewhere | 0.23(0.57) | 0.37(0.80) | **P<0.001** | 0.28 (0.67) | 0.21 (0.59) | 0.30 (0.65) | 0.22 (0.52) | 0.576 | 0.26 (0.64) | 0.18 (0.66) | 0.00 (0.00) | 0.38 (0.75) | 0.692 |
| 20. I forget my name or who I am | 0.04(0.32) | 0.07(0.44) | **0.043** | 0.07 (0.43) | 0.02 (0.19) | 0.00 (0.00) | 0.05 (0.31) | 0.251 | 0.05 (0.34) | 0.18 (0.85) | 0.00 (0.00) | 0.00 (0.00) | 0.307 |
| ***Note:*** *Values are shown as Mean (SD); Sig. represents the p-value, where p < 0.05 is bolded, indicating a statistically significant difference.* | | | | | | | | | | | | | |

| **Supplementary Table 4: Item-Level Distribution of WERCAP Dissociation Symptoms (N = 949)** | | | | |
| --- | --- | --- | --- | --- |
| **Item** | **Mean** | **SD** | **Skewness** | **Kurtosis** |
| 1. I usually spend _____ hours daily daydreaming about an imaginary life or event | 1.02 | 0.88 | 0.67 | 0.24 |
| 2. My daydreams feel very realistic, as if they are really happening | 1.31 | 1.22 | 0.48 | -0.73 |
| 3. When I daydream, it is very hard to work, study or complete tasks | 0.98 | 1.2 | 0.97 | -0.12 |
| 4. I use music or electronics to daydream | 0.95 | 1.26 | 1.02 | -0.21 |
| 5. I walk around, move my arms or make expressions with my face when I daydream | 0.82 | 1.22 | 1.3 | 0.5 |
| 6. I prefer daydreaming than spending time with others | 0.78 | 1.2 | 1.36 | 0.7 |
| 7. I feel like I am looking at myself from outside my body | 0.63 | 1.04 | 1.61 | 1.72 |
| 8. When I look at my hands or legs, they don’t feel like they belong to me | 0.22 | 0.69 | 3.52 | 12.74 |
| 9. When I see myself in the mirror, it feels like I am looking at someone else | 0.32 | 0.78 | 2.7 | 7.02 |
| 10. My thoughts, feelings or emotions don’t feel like they are mine | 0.45 | 0.84 | 1.84 | 2.76 |
| 11. I feel like I am two or more different people with different personalities | 0.73 | 1.14 | 1.42 | 0.97 |
| 12. People or things around me feel strange, like they are part of a dream, movie or video game | 0.43 | 0.88 | 2.17 | 4.15 |
| 13. It feels like I am separated from the world around me by a glass screen or transparent cloth | 0.25 | 0.69 | 3.17 | 10.45 |
| 14. It feels like time is moving much faster or slower than it should | 1.09 | 1.22 | 0.77 | -0.41 |
| 15. The colors or sizes of things around me seem different than they should be | 0.28 | 0.69 | 2.73 | 7.68 |
| 16. The sounds of people or things seem different than they should be | 0.31 | 0.73 | 2.53 | 6.19 |
| 17. I forget important or traumatic events that happened to me | 0.69 | 1.04 | 1.46 | 1.31 |
| 18. I forget conversations I had or things that I did | 0.86 | 1.06 | 1.01 | 0.19 |
| 19. I forget how I traveled and arrived somewhere | 0.26 | 0.64 | 2.69 | 7.33 |
| 20. I forget my name or who I am | 0.05 | 0.35 | 8.83 | 87.07 |
| Items were scored on a 0-4 scale | | | | |

| **Supplementary Table 5: The frequency of the dissociation items** | | | | | | | | | | | |
| --- | --- | --- | --- | --- | --- | --- | --- | --- | --- | --- | --- |
| Dissociation items | No | | Rarely | | Sometimes | | Often | | Almost Always | |  |
|  | Count | Percentage (%) | Count | Percentage (%) | Count | Percentage (%) | Count | Percentage (%) | Count | Percentage (%) | Percentage (%) |
| 1. I usually spend _____ hours daily daydreaming about an imaginary life or event | 286 | 30.3% | 410 | 43.4% | 199 | 21.1% | 41 | 4.3% | 9 | 1.0% | 26.4% |
| 2. My daydreams feel very realistic, as if they are really happening | 350 | 37.0% | 152 | 16.1% | 306 | 32.3% | 82 | 8.7% | 57 | 6.0% | 47.0% |
| 3. When I daydream, it is very hard to work, study or complete tasks | 483 | 51.1% | 161 | 17.0% | 188 | 19.9% | 68 | 7.2% | 46 | 4.9% | 32.0% |
| 4. I use music or electronics to daydream | 535 | 56.4% | 109 | 11.5% | 171 | 18.0% | 78 | 8.2% | 55 | 5.8% | 32.0% |
| 5. I walk around, move my arms or make expressions with my face when I daydream | 581 | 61.4% | 120 | 12.7% | 132 | 13.9% | 60 | 6.3% | 54 | 5.7% | 25.9% |
| 6. I prefer daydreaming than spending time with others | 603 | 63.6% | 103 | 10.9% | 140 | 14.8% | 52 | 5.5% | 50 | 5.3% | 25.6% |
| 7. I feel like I am looking at myself from outside my body | 640 | 67.5% | 113 | 11.9% | 132 | 13.9% | 35 | 3.7% | 28 | 3.0% | 20.6% |
| 8. When I look at my hands or legs, they don’t feel like they belong to me | 833 | 87.9% | 54 | 5.7% | 36 | 3.8% | 15 | 1.6% | 10 | 1.1% | 6.5% |
| 9. When I see myself in the mirror, it feels like I am looking at someone else | 780 | 82.6% | 71 | 7.5% | 62 | 6.6% | 20 | 2.1% | 11 | 1.2% | 9.9% |
| 10. My thoughts, feelings or emotions don’t feel like they are mine | 702 | 74.1% | 98 | 10.3% | 127 | 13.4% | 12 | 1.3% | 9 | 0.9% | 15.6% |
| 11. I feel like I am two or more different people with different personalities | 610 | 64.3% | 106 | 11.2% | 149 | 15.7% | 43 | 4.5% | 40 | 4.2% | 24.4% |
| 12. People or things around me feel strange, like they are part of a dream, movie or video game | 718 | 76.0% | 105 | 11.1% | 79 | 8.4% | 29 | 3.1% | 14 | 1.5% | 13.0% |
| 13. It feels like I am separated from the world around me by a glass screen or transparent cloth | 810 | 85.6% | 66 | 7.0% | 49 | 5.2% | 12 | 1.3% | 9 | 1.0% | 7.5% |
| 14. It feels like time is moving much faster or slower than it should | 450 | 47.5% | 124 | 13.1% | 266 | 28.1% | 52 | 5.5% | 56 | 5.9% | 39.5% |
| 15. The colors or sizes of things around me seem different than they should be | 783 | 82.7% | 85 | 9.0% | 63 | 6.7% | 10 | 1.1% | 6 | 0.6% | 8.4% |
| 16. The sounds of people or things seem different than they should be | 766 | 80.9% | 95 | 10.0% | 62 | 6.5% | 19 | 2.0% | 5 | 0.5% | 9.0% |
| 17. I forget important or traumatic events that happened to me | 584 | 61.7% | 167 | 17.6% | 126 | 13.3% | 45 | 4.8% | 25 | 2.6% | 20.7% |
| 18. I forget conversations I had or things that I did | 490 | 51.7% | 188 | 19.8% | 202 | 21.3% | 44 | 4.6% | 24 | 2.5% | 28.4% |
| 19. I forget how I traveled and arrived somewhere | 780 | 82.3% | 104 | 11.0% | 48 | 5.1% | 14 | 1.5% | 2 | 0.2% | 6.8% |
| 20. I forget my name or who I am | 921 | 97.2% | 16 | 1.7% | 6 | 0.6% | 0 | 0.0% | 5 | 0.5% | 1.1% |
| **Note:** *Percentages are based on valid responses (N = 949). The final column represents the combined prevalence of dissociative experiences, calculated as the sum of “Sometimes,” “Often,” and “Almost Always” responses for each item.* | | | | | | | | | | | |

| **Supplementary Table 6: Polychoric Correlations Among WERCAP Dissociation Items (N = 949)** | | | | | | | | | | | | | | | | | | | | | |
| --- | --- | --- | --- | --- | --- | --- | --- | --- | --- | --- | --- | --- | --- | --- | --- | --- | --- | --- | --- | --- | --- |
| **Dissociation Items** | 1 | 2 | 3 | 4 | 5 | 6 | 7 | 8 | 9 | 10 | 11 | 12 | 13 | 14 | 15 | 16 | 17 | 18 | 19 | 20 |  |
| 1 | 1.00 | 0.65 | 0.60 | 0.47 | 0.54 | 0.49 | 0.42 | 0.32 | 0.39 | 0.40 | 0.43 | 0.45 | 0.35 | 0.38 | 0.32 | 0.37 | 0.26 | 0.14 | 0.16 | 0.15 |  |
| 2 | 0.65 | 1.00 | 0.55 | 0.49 | 0.48 | 0.50 | 0.49 | 0.42 | 0.36 | 0.44 | 0.46 | 0.47 | 0.34 | 0.38 | 0.34 | 0.44 | 0.26 | 0.12 | 0.19 | 0.15 |  |
| 3 | 0.60 | 0.55 | 1.00 | 0.46 | 0.58 | 0.47 | 0.44 | 0.39 | 0.41 | 0.51 | 0.44 | 0.48 | 0.42 | 0.44 | 0.43 | 0.44 | 0.27 | 0.16 | 0.22 | 0.18 |  |
| 4 | 0.47 | 0.49 | 0.46 | 1.00 | 0.52 | 0.46 | 0.39 | 0.32 | 0.36 | 0.36 | 0.43 | 0.39 | 0.41 | 0.36 | 0.26 | 0.34 | 0.22 | 0.15 | 0.17 | 0.23 |  |
| 5 | 0.54 | 0.48 | 0.58 | 0.52 | 1.00 | 0.56 | 0.49 | 0.44 | 0.39 | 0.47 | 0.51 | 0.49 | 0.50 | 0.40 | 0.33 | 0.33 | 0.25 | 0.25 | 0.22 | 0.25 |  |
| 6 | 0.49 | 0.50 | 0.47 | 0.46 | 0.56 | 1.00 | 0.48 | 0.40 | 0.32 | 0.50 | 0.51 | 0.47 | 0.48 | 0.39 | 0.28 | 0.38 | 0.23 | 0.25 | 0.22 | 0.31 |  |
| 7 | 0.42 | 0.49 | 0.44 | 0.39 | 0.49 | 0.48 | 1.00 | 0.61 | 0.50 | 0.55 | 0.53 | 0.49 | 0.56 | 0.46 | 0.47 | 0.46 | 0.25 | 0.18 | 0.20 | 0.13 |  |
| 8 | 0.32 | 0.42 | 0.39 | 0.32 | 0.44 | 0.40 | 0.61 | 1.00 | 0.67 | 0.59 | 0.55 | 0.52 | 0.57 | 0.32 | 0.50 | 0.43 | 0.25 | 0.19 | 0.31 | 0.30 |  |
| 9 | 0.39 | 0.36 | 0.41 | 0.36 | 0.39 | 0.32 | 0.50 | 0.67 | 1.00 | 0.63 | 0.54 | 0.56 | 0.49 | 0.39 | 0.52 | 0.47 | 0.28 | 0.21 | 0.30 | 0.36 |  |
| 10 | 0.40 | 0.44 | 0.51 | 0.36 | 0.47 | 0.50 | 0.55 | 0.59 | 0.63 | 1.00 | 0.65 | 0.59 | 0.61 | 0.49 | 0.56 | 0.56 | 0.27 | 0.19 | 0.24 | 0.11 |  |
| 11 | 0.43 | 0.46 | 0.44 | 0.43 | 0.51 | 0.51 | 0.53 | 0.55 | 0.54 | 0.65 | 1.00 | 0.60 | 0.57 | 0.48 | 0.40 | 0.52 | 0.22 | 0.17 | 0.23 | 0.28 |  |
| 12 | 0.45 | 0.47 | 0.48 | 0.39 | 0.49 | 0.47 | 0.49 | 0.52 | 0.56 | 0.59 | 0.60 | 1.00 | 0.62 | 0.50 | 0.53 | 0.58 | 0.25 | 0.19 | 0.29 | 0.20 |  |
| 13 | 0.35 | 0.34 | 0.42 | 0.41 | 0.50 | 0.48 | 0.56 | 0.57 | 0.49 | 0.61 | 0.57 | 0.62 | 1.00 | 0.59 | 0.55 | 0.60 | 0.29 | 0.22 | 0.30 | 0.32 |  |
| 14 | 0.38 | 0.38 | 0.44 | 0.36 | 0.40 | 0.39 | 0.46 | 0.32 | 0.39 | 0.49 | 0.48 | 0.50 | 0.59 | 1.00 | 0.56 | 0.57 | 0.37 | 0.30 | 0.21 | 0.11 |  |
| 15 | 0.32 | 0.34 | 0.43 | 0.26 | 0.33 | 0.28 | 0.47 | 0.50 | 0.52 | 0.56 | 0.40 | 0.53 | 0.55 | 0.56 | 1.00 | 0.72 | 0.25 | 0.25 | 0.24 | 0.15 |  |
| 16 | 0.37 | 0.44 | 0.44 | 0.34 | 0.33 | 0.38 | 0.46 | 0.43 | 0.47 | 0.56 | 0.52 | 0.58 | 0.60 | 0.57 | 0.72 | 1.00 | 0.32 | 0.24 | 0.30 | 0.08 |  |
| 17 | 0.26 | 0.26 | 0.27 | 0.22 | 0.25 | 0.23 | 0.25 | 0.25 | 0.28 | 0.27 | 0.22 | 0.25 | 0.29 | 0.37 | 0.25 | 0.32 | 1.00 | 0.32 | 0.26 | 0.13 |  |
| 18 | 0.14 | 0.12 | 0.16 | 0.15 | 0.25 | 0.25 | 0.18 | 0.19 | 0.21 | 0.19 | 0.17 | 0.19 | 0.22 | 0.30 | 0.25 | 0.24 | 0.32 | 1.00 | 0.50 | 0.28 |  |
| 19 | 0.16 | 0.19 | 0.22 | 0.17 | 0.22 | 0.22 | 0.20 | 0.31 | 0.30 | 0.24 | 0.23 | 0.29 | 0.30 | 0.21 | 0.24 | 0.30 | 0.26 | 0.50 | 1.00 | 0.60 |  |
| 20 | 0.15 | 0.15 | 0.18 | 0.23 | 0.25 | 0.31 | 0.13 | 0.30 | 0.36 | 0.11 | 0.28 | 0.20 | 0.32 | 0.11 | 0.15 | 0.08 | 0.13 | 0.28 | 0.60 | 1.00 |  |
| Polychoric correlations among 20 WERCAP dissociation items. Values above 0.3 indicate moderate correlations | | | | | | | | | | | | | | | | | | | | | |

| **Supplementary Table 7: KMO and Bartlett’s Test Results for Factor Analysis** | | | |
| --- | --- | --- | --- |
| **Test** | **Statistic** | **Value** | **Interpretation** |
| **Kaiser-Meyer-Olkin (KMO) Measure of Sampling Adequacy** | — | 0.913 | **Excellent** |
| **Bartlett’s Test of Sphericity** | Approx. Chi-Square | 5558.527 | — |
|  | df | 190 | — |
|  | **Sig.** | **<0.001** | **factorable** |
|  | | | |

*The Kaiser–Meyer–Olkin (KMO) measure indicated excellent sampling adequacy (KMO = 0.911), and Bartlett’s Test of Sphericity was significant (χ²(190) = 5453.93, p < .001), confirming that the data were appropriate for factor analysis.*

|  | **Supplementary Table 8: Association Between Bipolar(a_WERCAP) and Dissociative Experiences** | | | | | | | | | | | | |
| --- | --- | --- | --- | --- | --- | --- | --- | --- | --- | --- | --- | --- | --- |
|  |  | | **No** | | **Rarely** | | **Sometimes** | **Often** | | **Almost Always** | | **Sig.** | |
|  | **Item** | | **Mean (Std. Deviation)** | | **Mean (Std. Deviation)** | | **Mean (Std. Deviation)** | **Mean (Std. Deviation)** | | **Mean (Std. Deviation)** | |  | |
|  | 1. I usually spend _____ hours daily daydreaming about an imaginary life or event | | 15.61 (8.35) | | 15.38 (8.57) | | 15.25 (8.64) | 14.12 (8.56) | | 15.14 (7.90) | | 0.72 | |
|  | 2. My daydreams feel very realistic, as if they are really happening | | 15.58 (15.32) | | 15.73 (15.05) | | 13.90 (15.22) | 16.53 (14.74) | | 14.85 (16.58) | | 0.105 | |
|  | 3.When I daydream, it is very hard to work, study or complete tasks | | 8.16 (15.20) | | 8.78 (14.68) | | 8.95 (15.94) | 8.36 (15.13) | | 9.43 (16.22) | | 0.788 | |
|  | 4.I use music or electronics to daydream | | 8.38 (15.37) | | 8.98 (14.73) | | 8.22 (15.19) | 8.69 (15.56) | | 8.70 (15.56) | | 0.712 | |
|  | 5.I walk around, move my arms or make expressions with my face when I daydream | | 8.31 (15.55) | | 8.98 (16.00) | | 8.15 (13.76) | 9.65 (14.31) | | 9.03 (15.00) | | 0.96 | |
|  | 6.I prefer daydreaming than spending time with others | | 8.37 (15.39) | | 9.64 (13.61) | | 8.20 (16.61) | 7.60 (12.00) | | 7.05 (16.80) | | 0.182 | |
|  | 7. I feel like I am looking at myself from outside my body | | 8.51 (15.45) | | 7.36 (13.48) | | 8.73 (15.68) | 7.64 (14.25) | | 9.26 (12.91) | | 0.223 | |
|  | 8.When I look at my hands or legs, they don’t feel like they belong to me | | 8.49 (15.54) | | 7.70 (14.84) | | 8.64 (14.37) | 8.27 (15.00) | | 8.38 (14.33) | | 0.304 | |
|  | 9.When I see myself in the mirror, it feels like I am looking at someone else | | 8.43 (15.24) | | 8.98 (15.90) | | 8.29 (14.82) | 7.85 (16.49) | | 9.22 (15.05) | | 0.64 | |
|  | 10.My thoughts, feelings or emotions don’t feel like they are mine | | 8.29 (15.41) | | 8.66 (13.95) | | 9.45 (15.73) | 7.31 (15.72) | | 7.89 (16.86) | | 0.75 | |
|  | 11. I feel like I am two or more different people with different personalities | | 8.41 (15.30) | | 8.58 (15.27) | | 8.43 (16.08) | 9.44 (9.50) | | 8.80 (17.89) | | 0.472 | |
|  | 12. People or things around me feel strange, like they are part of a dream, movie or video game | | 8.44 (15.50) | | 8.11 (14.39) | | 9.14 (15.15) | 8.22 (15.10) | | 7.17 (16.46) | | 0.141 | |
|  | 13.It feels like I am separated from the world around me by a glass screen or transparent cloth | | 8.58 (15.23) | | 7.27 (14.80) | | 8.47 (16.38) | 9.45 (17.10) | | 8.97 (16.00) | | 0.581 | |
|  | 14.It feels like time is moving much faster or slower than it should | | 8.28 (15.18) | | 9.39 (14.39) | | 9.67 (17.81) | 8.60 (15.26) | | 4.60 (16.40) | | 0.76 | |
|  | 15.The colors or sizes or things around me seem different than they should be | | 8.42 (15.36) | | 7.71 (14.85) | | 9.56 (15.21) | 8.67 (15.40) | | 10.01 (16.32) | | 0.147 | |
|  | 16.The sounds of people or things seem different than they should be | | 8.09 (14.78) | | 8.87 (15.50) | | 9.52 (16.09) | 8.56 (16.48) | | 8.55 (15.33) | | 0.927 | |
|  | 17. Forget important or traumatic events that happened to me | | 8.19 (15.08) | | 8.69 (16.63) | | 9.11 (15.94) | 7.84 (15.21) | | 7.10 (16.00) | | 0.331 | |
|  | 18.I forget conversations I had or things that I did | | 8.33 (15.24) | | 9.15 (17.50) | | 8.58 (17.50) | 8.62 (16.00) | | 21.21 (15.29) | | 0.49 | |
|  | 19.I forget how I traveled and arrived somewhere | | 8.38 (16.63) | | 11.87 (15.94) | | 10.93 (15.21) | 9.14 (15.08) | | 8.46 (16.63) | | 0.667 | |
|  | 20.I forget my name or who I am | | 9.15 (16.08) | | 8.58 (9.14) | | 8.62 (15.30) | 9.15 (7.17) | | 15.21 (16.08) | | 0.49 | |
|  | **Note:** *Values are presented as mean (standard deviation) of a-WERCAP scores across frequencies of dissociative experiences. Significance values are based on one-way ANOVA, with p < .05 considered statistically significant.* | | | | | | | | | | | | |
| **Supplementary Table 9: Association Between Dissociative Experiences Factors and Bipolar(a_WERCAP)** | | | | | | | | | | | | |  |
|  | | a_WERCAP Score | | Dissociation scores | | Perceptual distortion | | | Daydream Intensity | | Memory lapses | |  |
| a_WERCAP Score | | 1 | | 0.009 | | -0.012 | | | -0.009 | | 0.047 | |  |
| Dissociation scores | | 0.009 | | 1 | | .845^**^ | | | .891^**^ | | .583^**^ | |  |
| Perceptual distortion | | -0.012 | | .845^**^ | | 1 | | | .618^**^ | | .444^**^ | |  |
| Daydream Intensity | | -0.009 | | .891^**^ | | .618^**^ | | | 1 | | .331^**^ | |  |
| Memory lapses | | 0.047 | | .583^**^ | | .444^**^ | | | .331^**^ | | 1 | |  |
| **Note***: Pearson correlation coefficients were used to assess the relationships between bipolar affective symptoms (a-WERCAP) and dissociative experience factors. Correlations marked with ** are statistically significant at the 0.01 level (two-tailed).* | | | | | | | | | | | | |  |
