# Supplementary material for: Dissociative experiences among Kenyan youth in the Nairobi metropolitan area: Associations with psychosis risk symptoms
Source: Glob Ment Health (Camb). 2026 Jun 25;13:e158. doi: 10.1017/gmh.2026.10265 (PMC13433326; doi:10.1017/gmh.2026.10265)
Supplement: Peer review history

# Reviewer report: Review: Dissociative experiences among Kenyan youth in the Nairobi metropolitan area: Associations with psychosis risk symptoms — R0/PR2

**DOI:** 10.1017/gmh.2026.10265.pr2

**Authors:** Mohamed Aden Hillow

**Affiliations:** PSYCHIATRY, Mathari National Teaching and Referral Hospital, Kenya

Overall Impression

This is an important and timely study addressing dissociative experiences among Kenyan youth and their relationship with psychosis risk. The manuscript is novel, well-powered (n=949), and highly relevant to global mental health, particularly in LMIC settings.

However, there are several conceptual, methodological, and interpretative issues that should be addressed to strengthen the manuscript prior to publication.

Major Comments

1. Conceptual Clarity: Dissociation vs Psychosis Risk

The manuscript oscillates between positioning dissociation as:

(a) an independent construct

(b) a risk marker for psychosis

(c) a potential prodromal feature

However, these are distinct theoretical positions, and the paper does not clearly commit to one.

Recommendation:

- Clearly define the conceptual model: Is dissociation a predictor, correlate, or overlapping phenotype? The discussion should avoid causal language given the cross-sectional design.

2. Contradictory Findings. There is a major internal inconsistency:

You report significant differences between high-risk vs low-risk groups (p < 0.001)!

But also, No correlation between dissociation and psychosis severity (r ≈ 0)

This is a critical issue that must be addressed.

Recommendation:

Provide an explanation for: Why categorical differences exist without dimensional correlation

Consider:

- Non-linear relationships

- Threshold effects

- Measurement limitations of WERCAP

3. Validity of the WERC-DS Tool

The WERC-DS is presented as “comprehensive,” “phenomenologically precise,” and “cross-cultural” But, there is limited evidence provided on validation in this population

Recommendation:

Clarify:

- Has WERC-DS been validated in Kenya or SSA? Provide Construct validity, Convergent validity (e.g., vs DES-II)

- The poor reliability of the memory subscale (α = 0.48) is concerning and under-discussed.

4. Sampling and Representativeness

The sample is described as “community-based,” but majority are Students, Unemployed, Urban/peri-urban. This limits generalizability.

Recommendation:

- Acknowledge this more explicitly. This is closer to a student-heavy convenience sample.

- Avoid statements implying national representativeness

5. Overinterpretation of Findings

- Several conclusions are too strong for cross-sectional data, for example: “early vulnerability marker” “precursor to psychosis”

Recommendation:

- Tone down causal language.

Replace with:

- “associated with”

- “may indicate”

Emphasize the need for longitudinal validation (already mentioned but should be stronger)

6. Wealth Index Findings – Interpretation Problem

The finding that higher wealth is associated with more dissociation contradicts existing literature. The explanation provided is internally inconsistent (you revert back to poverty-based explanations).

Recommendation:

Re-evaluate interpretation: Could this reflect Urban stress? Academic pressure? Reporting bias? This section needs clearer, logically consistent reasoning.

7. Trauma – Major Missing Variable

Dissociation is strongly linked to trauma, yet Trauma was not measured! This is a major limitation, not a minor one.

Recommendation:

Upgrade this to a central limitation. Avoid trauma-based interpretations in discussion unless measured.

Minor Comments

1. Clarity in Methods

- Clarify: Sampling strategy (random vs convenience). How “community” participants were identified.

- Explain why PCA + Varimax was used instead of more robust methods (e.g., CFA)

2. Factor Analysis

- You used PCA (not true EFA). This should be clarified or justified.

3. Memory Subscale Issue

- α = 0.48 suggests poor internal consistency. Consider revising or removing this subscale or clearly justify retaining it.

4. Terminology

- “Bipolar” vs “mood disorder risk” used interchangeably → clarify “Psychosis risk” vs “psychotic experiences” → distinguish clearly

# Reviewer report: Review: Dissociative experiences among Kenyan youth in the Nairobi metropolitan area: Associations with psychosis risk symptoms — R0/PR3

**DOI:** 10.1017/gmh.2026.10265.pr3

This manuscript examines the prevalence and structure of dissociative experiences (e.g., daydreaming, perceptual distortions, and memory lapses) among Kenyan youth aged 14–30 in the Nairobi Metropolitan Area, and explores their associations with sociodemographic factors and risk for psychosis and mood disorders. Using cross-sectional survey data and factor analysis of the WERC-DS, the study identifies three key dimensions of dissociation and finds that these experiences particularly immersive daydreaming and perceptual distortions are common and significantly elevated among individuals at high risk for psychosis, but not associated with mood disorder risk. The manuscript contributes to the existing literature by providing rare population-based evidence from an African context, highlighting the multidimensional nature of dissociation in non-Western youth populations, and suggesting dissociative experiences as potential markers of psychosis risk in low-resource settings.

Comments

1. The claim that this is the “first in the world” study providing such detailed data should be clarified and better justified in relation to existing global literature.

2. Dissociation is presented both as an independent construct and as a potential early marker of psychosis; please clarify the theoretical position and which framework is primary to the interpretation.

3. Given the cross-sectional design, please clarify how over-interpretation of dissociation as an “early vulnerability marker” was avoided, in the absence of temporal evidence.

4. There appears to be an inconsistency between significant group differences by psychosis risk and near-zero correlations with psychotic symptom severity; this requires clarification and discussion of implications.

5. The Memory Lapses factor shows low reliability (α = 0.48); please clarify whether it is sufficiently robust to retain and interpret.

6. Trauma is discussed extensively in the introduction but was not measured; please clarify how this omission affects interpretation of dissociation mechanisms.

7. The finding that higher wealth is associated with higher dissociation contrasts with prior literature; possible explanations (e.g., measurement, confounding, contextual factors) should be elaborated.

8. Since participants were mainly recruited from educational institutions and urban settings, please clarify the generalizability of findings to non-student and rural youth populations.

9. Exploratory factor analysis was used; please clarify why confirmatory approaches (e.g., CFA) were not considered to validate the factor structure.

10. Data collection spanned 2021–2025; please indicate whether temporal or contextual influences (e.g., COVID-19) were considered.

Main suggestions for improvement

1. The authors are encouraged to include a conceptual model or figure illustrating the proposed relationship between dissociative experiences and psychosis risk to strengthen theoretical clarity.

2. The authors are encouraged to conduct or report sensitivity analyses (e.g., by age group, or student vs. community samples) to better understand subgroup variability.

3. The authors should include a clearer discussion distinguishing normative vs. clinically significant dissociative experiences, particularly given the high prevalence of daydreaming reported.

# Reviewer report: Review: Dissociative experiences among Kenyan youth in the Nairobi metropolitan area: Associations with psychosis risk symptoms — R0/PR4

**DOI:** 10.1017/gmh.2026.10265.pr4

While this research project clearly has some merit, there are significant flaws in the research design and the research questions/aims cannot be adequately addressed by the instruments used, particularly as this is a cross-sectional study.

The first aim CAN be adequately addressed by the research design: “To identify and describe the patterns of dissociative experiences, including perceptual distortions, immersive daydreaming, and memory lapses, among your aged 14-30 years.”

But even here there are two major challenges -- with the sample and with the instrument. First of all, how representative is the sample of Kenyan youth in the Nairobi area? Not enough is said about how the sample was generated, including how participants were solicited, and how many were eliminated. Secondly, how valid a measure of dissociation in the Washington Early Recognition Center Dissociation Screen? There are a number of well-established dissociation instruments -- how well does the WERC-DS correlate with those? That data needs to be reported or at least summarized before the reader can assume that the WERC-DS is a valid measure of dissociation.

The goal of using a dissociation screen to predict psychosis-proneness is also problematic. First of all, the study is entirely cross-sectional and the criterion for psychosis is not any clinical diagnosis or assessment but self-report on a psychosis screen instrument generated by the same team as the dissociation screen. Overlap between these psychosis and dissociation instruments is not unexpected for that reason and also because at least three items on the WERCAP have been conceptualized as dissociative in nature in clinical and empirical research and diagnostic systems -- voice hearing and passive influence symptoms. Secondly, the authors provide no foundation for this hypothesis. There is extensive literature on the relationship between dissociation and psychosis which could support a supposition that dissociation could predict psychotic symptoms, but the authors do not avail themselves of any of this. And finally, the most likely diagnoses or clinical syndromes to be related to dissociation would be dissociative disorders. Why do the authors neglect this obvious prediction in favor of psychotic and mood symptoms?

Though there are no references to extensive dissociative disorders, there are extensive references to a proposed dissociative disorder, maladaptive daydreaming. As far as this reviewer is aware, however, there is limited or no research suggesting that maladaptive daydreaming may predict psychosis-proneness. If there is, the authors would be advised to discuss it.

The strongest part of the article involves the factor analysis of items on the WERC-DS. As this does not yet appear to be an established instrument (it is not listed as a screening instrument on the Washington Early Recognition Center website), this research might be best served to establish cross-cultural validity of this instrument and compare the factor loadings with prior research using the same instrument. This could be a profitable use of this data even if there are questions about the generalizability of the sample.

In sum, while the research study itself appears to have some worth, the aims the researchers have provided -- particularly that of predicting psychosis proneness -- cannot be met with this research design. The authors are advised to rather use the data to buttress the cross-cultural validity and reliability of this new instrument. More data, however, on its relation to older, established dissociation instruments need to be provided before it can be accepted as a valid dissociation screening instrument.

# Editor report: Recommendation: Dissociative experiences among Kenyan youth in the Nairobi metropolitan area: Associations with psychosis risk symptoms — R0/PR5

**DOI:** 10.1017/gmh.2026.10265.pr5

**Affiliations:** Centre for Rural Health, University of KwaZulu-Natal, South Africa

Dear Prof Ndetei,

Following inputs from two independent reviewers, a decision has been reached for your submission titled “Dissociative Experiences among Kenyan Youth in the Nairobi Metropolitan Area: Associations with Psychosis Risk Symptoms”. The recommended outcome from the review was “Major Revision”. Please read reviewer comments carefully, and address each point, while documenting your response to each point raised before re-submitting your manuscript. We are looking forward to receiving your revised submission.

Sincerely,

Prof. André Janse van Rensburg

Cambridge Prisms: Global Mental Health

# Editor report: Decision: Dissociative experiences among Kenyan youth in the Nairobi metropolitan area: Associations with psychosis risk symptoms — R0/PR6

**DOI:** 10.1017/gmh.2026.10265.pr6

**Affiliations:** London School of Hygiene & Tropical Medicine, United Kingdom of Great Britain and Northern Ireland

No accompanying comment.

# Reviewer report: Review: Dissociative experiences among Kenyan youth in the Nairobi metropolitan area: Associations with psychosis risk symptoms — R1/PR8

**DOI:** 10.1017/gmh.2026.10265.pr8

**Authors:** Mohamed Aden Hillow

**Affiliations:** PSYCHIATRY, Mathari National Teaching and Referral Hospital, Kenya

The revised manuscript has improved substantially and adequately addresses the major concerns raised in the initial review. The authors have clarified the conceptual framing of dissociation, appropriately moderated causal interpretations, strengthened the discussion of psychosis-risk associations, and improved transparency regarding sampling limitations and the exploratory nature of the psychometric analyses.

The study represents an important and novel contribution to the literature on dissociative experiences and psychosis-risk phenomena among African youth populations. The large community-based sample and focus on culturally relevant mental health constructs add significant value to the global mental health field.

While a few minor editorial and formatting inconsistencies remain in the proof version, these do not substantially affect the scientific quality or interpretation of the findings and can be addressed during copyediting.

Overall, I believe the manuscript is suitable for publication in its current revised form.

# Reviewer report: Review: Dissociative experiences among Kenyan youth in the Nairobi metropolitan area: Associations with psychosis risk symptoms — R1/PR9

**DOI:** 10.1017/gmh.2026.10265.pr9

1. The abstract contains duplicated “Background” and “Conclusions” sections. Could the authors carefully review the abstract for repeated text introduced during revision or proof preparation?

2. The manuscript states that this is “the first in the world” to provide detailed data on dissociative symptoms and their relationship with psychosis and mood disorders. Could the authors moderate this claim, as it may be difficult to substantiate fully?

3. The findings regarding psychosis associations require clarification. High-risk participants reportedly had significantly higher dissociation scores, yet later analyses indicate no meaningful correlations between psychotic experience severity and dissociation scores. Could the authors clarify the distinction between categorical risk-group comparisons and continuous correlation analyses?

4. The Memory Lapses subscale demonstrated poor internal consistency (α = 0.48). Could the authors discuss more explicitly how this low reliability may affect interpretation of findings related to this subscale?

5. In several sections, maladaptive daydreaming is discussed as a possible vulnerability marker for psychosis. Given the cross-sectional design and absence of clinical diagnostic assessment, could the authors ensure that interpretations remain cautious and associative rather than predictive or causal?

6. The manuscript would benefit from clearer explanation of how dissociative experiences differ phenomenologically from psychotic symptoms, particularly because several items involve perceptual distortions that may conceptually overlap with psychosis-risk experiences.

7. Several typographical and formatting issues remain in the revised manuscript including duplicated text, spacing inconsistencies, and typographical errors. A final careful proofreading and language edit is recommended before publication.

# Editor report: Recommendation: Dissociative experiences among Kenyan youth in the Nairobi metropolitan area: Associations with psychosis risk symptoms — R1/PR10

**DOI:** 10.1017/gmh.2026.10265.pr10

**Affiliations:** Centre for Rural Health, University of KwaZulu-Natal, South Africa

Dear Prof Ndetei

Following further review of your submission titled “Dissociative Experiences among Kenyan Youth in the Nairobi Metropolitan Area: Associations with Psychosis Risk Symptoms”, minor revisions have been recommended. 

Please read reviewer comments carefully, and address each point, while documenting your response to each point raised before re-submitting your manuscript. We are looking forward to receiving your revised submission.

Best regards,

Prof André J van Rensburg

# Editor report: Decision: Dissociative experiences among Kenyan youth in the Nairobi metropolitan area: Associations with psychosis risk symptoms — R1/PR11

**DOI:** 10.1017/gmh.2026.10265.pr11

**Affiliations:** London School of Hygiene & Tropical Medicine, United Kingdom of Great Britain and Northern Ireland

No accompanying comment.

# Editor report: Recommendation: Dissociative experiences among Kenyan youth in the Nairobi metropolitan area: Associations with psychosis risk symptoms — R2/PR13

**DOI:** 10.1017/gmh.2026.10265.pr13

**Affiliations:** Centre for Rural Health, University of KwaZulu-Natal, South Africa

Dear Prof Ndetei,

Following your revisions in response to peer review critiques and inputs, we have the pleasure to inform you that your paper has been accepted for publication. The editorial office will be in touch with next steps shortly.

Sincerely,

Prof. André Janse van Rensburg

Cambridge Prisms: Global Mental Health

# Editor report: Decision: Dissociative experiences among Kenyan youth in the Nairobi metropolitan area: Associations with psychosis risk symptoms — R2/PR14

**DOI:** 10.1017/gmh.2026.10265.pr14

**Affiliations:** London School of Hygiene & Tropical Medicine, United Kingdom of Great Britain and Northern Ireland

No accompanying comment.
